# Capturing a glycosylase reaction intermediate in DNA repair by freeze-trapping of a pH-responsive hOGG1 mutant

**DOI:** 10.1093/nar/gkaf718

**Published:** 2025-08-04

**Authors:** Masaki Unno, Masayuki Morikawa, Vladimír Sychrovský, Masataka Koga, Nozomi Minowa, Saki Komuro, Mami Shimizu, Mariko Fukuta, Fuuka Tsuyuguchi, Haruka Mano, Yusuke Ochi, Katsuyuki Nakashima, Yasuko Okamoto, Tomohide Saio, Yoshikazu Hattori, Yoshiyuki Tanaka

**Affiliations:** Graduate School of Science and Engineering, Ibaraki University, Hitachi, Ibaraki 316-8511, Japan; Research and Education Center for Atomic Sciences, Ibaraki University, Naka-Tokai, Ibaraki 319-1106, Japan; Laboratory of Analytical Chemistry, Faculty of Pharmaceutical Sciences, Tokushima Bunri University, Yamashiro-cho, Tokushima 770-8514, Japan; Institute of Organic Chemistry and Biochemistry, Czech Academy of Sciences, Flemingovo náměstí 2, Praha, 160 00, Czech Republic; Graduate School of Science and Engineering, Ibaraki University, Hitachi, Ibaraki 316-8511, Japan; Graduate School of Science and Engineering, Ibaraki University, Hitachi, Ibaraki 316-8511, Japan; Graduate School of Science and Engineering, Ibaraki University, Hitachi, Ibaraki 316-8511, Japan; Graduate School of Science and Engineering, Ibaraki University, Hitachi, Ibaraki 316-8511, Japan; Laboratory of Analytical Chemistry, Faculty of Pharmaceutical Sciences, Tokushima Bunri University, Yamashiro-cho, Tokushima 770-8514, Japan; Laboratory of Analytical Chemistry, Faculty of Pharmaceutical Sciences, Tokushima Bunri University, Yamashiro-cho, Tokushima 770-8514, Japan; Laboratory of Analytical Chemistry, Faculty of Pharmaceutical Sciences, Tokushima Bunri University, Yamashiro-cho, Tokushima 770-8514, Japan; Laboratory of Analytical Chemistry, Faculty of Pharmaceutical Sciences, Tokushima Bunri University, Yamashiro-cho, Tokushima 770-8514, Japan; Laboratory of Analytical Chemistry, Faculty of Pharmaceutical Sciences, Tokushima Bunri University, Yamashiro-cho, Tokushima 770-8514, Japan; Laboratory of Analytical Chemistry, Faculty of Pharmaceutical Sciences, Tokushima Bunri University, Yamashiro-cho, Tokushima 770-8514, Japan; Institute of Advanced Medical Sciences, Tokushima University, Kuramoto-cho 3-18-15, Tokushima 770-8503, Japan; Laboratory of Analytical Chemistry, Faculty of Pharmaceutical Sciences, Tokushima Bunri University, Yamashiro-cho, Tokushima 770-8514, Japan; Institute of Advanced Medical Sciences, Tokushima University, Kuramoto-cho 3-18-15, Tokushima 770-8503, Japan; Laboratory of Analytical Chemistry, Faculty of Pharmaceutical Sciences, Tokushima Bunri University, Yamashiro-cho, Tokushima 770-8514, Japan

## Abstract

The human 8-oxoguanine DNA glycosylase 1 (hOGG1) is a bifunctional DNA repair enzyme that possesses both glycosylase and AP-lyase activity. Its AP-lyase reaction mechanism had been revealed by crystallographic capturing of the intermediate adduct. However, no intermediate within the glycosylase reaction was reported to date and the relevant reaction mechanism thus remained unresolved. In this work, we studied the glycosylase reaction of hOGG1 by time-resolved crystallography and spectroscopic/enzymological analyses. To trigger the glycosylase reaction within a crystal, we created a pH-responsive mutant of hOGG1 in which lysine 249 (K249) has been replaced by histidine (H), and designated hOGG1(K249H). Using hOGG1(K249H), a reactive intermediate state of the hOGG1(K249H)–DNA complex was captured in crystal upon pH activation. An unprecedented, ribose-ring-opened hemiaminal structure at the 8-oxoguanine (oxoG) site was found. Based on the structure of the reaction intermediate and QM/MM (quantum mechanics/molecular mechanics) calculations, a glycosylase reaction pathway of hOGG1(K249H) was identified where the aspartic acid 268 (D268) acts as a proton donor to O4′ of oxoG. Moreover, enzymologically derived p*K*_a_ (4.5) of a catalytic residue indicated that the observed p*K*_a_ can be attributed to the carboxy group of D268. Thus, a reaction mechanism of the glycosylase reaction by hOGG1(K249H) has been proposed.

## Graphical Abstract

**Graphical Abstract:**
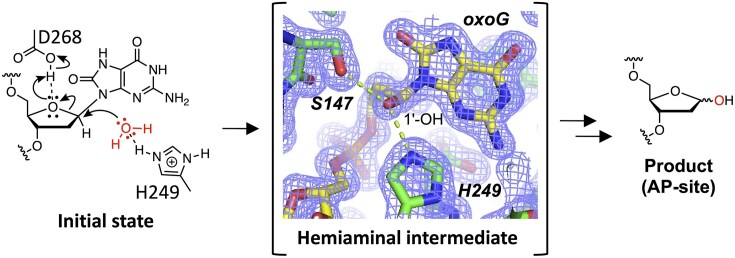

## Introduction

**Figure 1.**
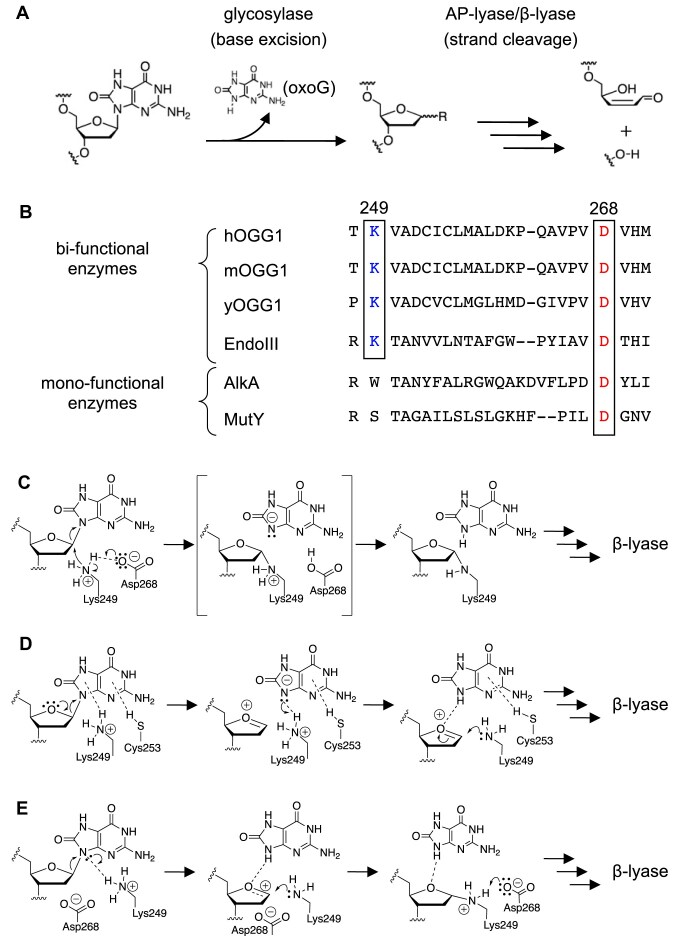
Reaction mechanism of hOGG1 and sequence alignment of related proteins. (**A**) Overall reaction pathway and the respective functions of hOGG1 (glycosylase and AP-lyase activities). (**B**) Sequence alignment of hOGG1 and related proteins. They are classified into monofunctional/bifunctional groups. Highly conserved residues in hOGG1 (K249 and D268) are boxed. (**C**) Mechanism that was proposed elsewhere [13]. (**D**) Mechanism that was proposed in [9]. (**E**) Mechanism proposed by authors of [15]. In panels (C)–(E), only the glycosylase step is shown.

Deciphering the mechanism of DNA repair enzymes’ action can provide not only how genetic integrity is maintained [1] but also how the enzyme is related to the pathology of cancer [1–3], neurodegeneration [4–6], and so on. Among DNA repair enzymes, human 8-oxoguanine glycosylase 1 (hOGG1) plays a central role in the base excision repair (BER) system acting against the strongly mutagenic 7,8-dihydro-8-oxoguanine (oxoG) (Fig. 1) [1–3].

Generally, glycosylases are categorized into two groups, namely, monofunctional enzymes with glycosylase activity alone and bifunctional ones with both glycosylase and AP-lyase activity (Fig. 1B) [7, 8]. As apparent from the sequence alignment of glycosylase proteins (Fig. 1B), aspartic acid is preserved across the two categories, whereas lysine is preserved in the bifunctional enzymes [8–12]. In the case of hOGG1, Lys249 (K249) and Asp268 (D268) are conserved because it belongs to the bifunctional enzyme. As mutation of K249 to glutamine (K249Q) and D268 to asparagine (D268N) yielded inactive mutants of hOGG1 [7, 9, 13, 14], these two conserved residues are the catalytic ones.

Crystal structure of the inactive mutant proteins hOGG1(K249Q) and hOGG1(D268N) was determined in a complex with their substrate DNA, and possible catalytic mechanisms of hOGG1 were previously proposed (Fig. 1C and D) [9, 13]. Our theoretical modeling was related to the later mechanism (Fig. 1E) [15]. A number of concerns are yet to be addressed, such as the chemical role of the conserved residues, K249 and D268. To this end, the reaction pathway of hOGG1 must be determined, and capturing the reactive intermediate by crystallography is the ultimate goal [16–20]. In this way, the reaction pathway of heme oxygenase was studied [17–20], and the very existence of the Schiff base intermediate in the AP-lyase reaction of hOGG1 was demonstrated by trapping the hOGG1–substrate covalent adduct via the K249 residue [16]. To capture the intermediate state of the glycosylase reaction (while preventing the AP-lyase reaction), the AP-lyase responsive K249 residue [16] must be substituted by another residue such as histidine that is chemically similar (a basic amino acid) but at the same time features a significantly different nucleophilicity for the suppression of the AP-lyase activity (monofunctionalization). A monofunctionalized hOGG1 has already been reported [21]. However, the hOGG1 mutant for time-resolved crystallography satisfies an additional requirement. To initiate the glycosylase reaction at the desired timing in the crystal, the mutant whose activity can be triggered with an external stimulus is required.

The aim of the present study was to construct a monofunctional hOGG1 mutant that can be triggered with an external stimulus, and to capture the reactive intermediate by using the corresponding mutant. An enzymatic assay of the newly designed hOGG1(K249H) mutant in which the original K249 residue is replaced with histidine (H) revealed that the mutant protein exhibits enzymatic activity under acidic pH. Electrospray ionization mass spectrometry (ESI-MS) and NaBH_4_-reductive trapping of the Schiff base intermediate were employed to verify that hOGG1(K249H) surely lacked the AP-lyase activity. To capture the reaction intermediate, the crystal structures of the hOGG1(K249H)–substrate DNA complex before and after the enzymatic reaction were determined with time-resolved crystallography, employing the pH-triggered hOGG1(K249H) mutant. A ribose-ring-opened hemiaminal intermediate was captured, and a reaction pathway of hOGG1(K249H) was proposed and verified by quantum mechanics/molecular mechanics (QM/MM) calculations.

## Materials and methods

### Preparation of substrate DNA and hOGG1

Preparations of substrate DNA and hOGG1 are described in Supplementary Materials and Methods.

### Assay of enzymatic reaction of hOGG1

Enzymatic assay of wild-type hOGG1 and hOGG1(K249H) was performed in a solution containing 50 mM citric acid–Na_2_HPO_4_ buffer (pH 6.0), 100 mM NaCl, 5.0 μM DNA duplex F-oG23•C23, and 10 μM hOGG1 protein for 2 h at 15°C (see Supplementary Materials and Methods for the sequences of F-oG23 and C23). The reaction mixtures were split into two aliquots. One of them (8.0 μl) was mixed with 4.0 μl of the quench solution (300 mM NaOH, 30 mM EDTA), and incubated at 90°C for 5 min to convert the AP-site-containing DNA strand into the AP-lyase products [21]. The other aliquot (8.0 μl) was not treated with the quench solution to preserve the AP-site-containing DNA strand [21].

The enzymatic assay of hOGG1(K249H) was performed for 60 min at 15°C at a series of pH values in a solution containing 50 mM citric acid–Na_2_HPO_4_ buffer (pH 3.0–8.0), 100 mM NaCl, 5.0 μM DNA duplex F-oG23•C23, and 10 μM hOGG1(K249H). The reaction mixture (8.0 μl) was mixed with 2.0 μl of the quench solution (300 mM NaOH, 30 mM EDTA), and incubated at 90°C for 5 min.

**Figure 2.**
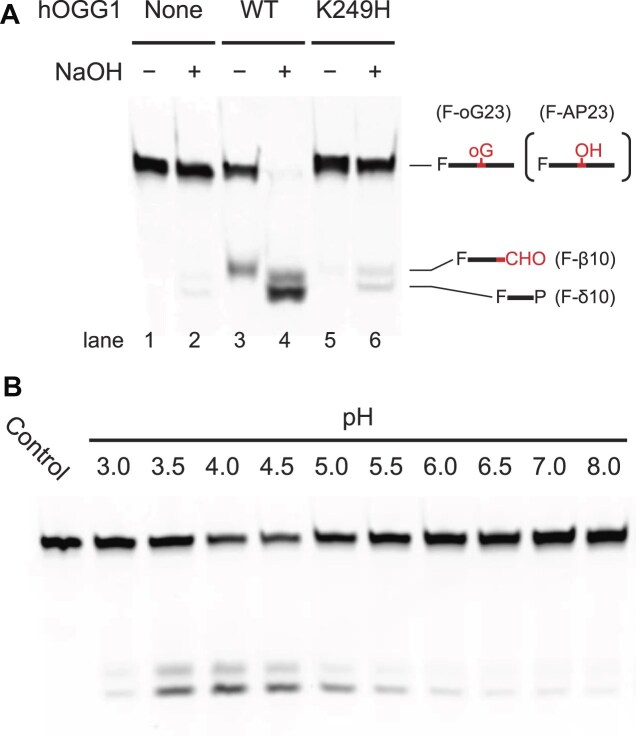
Assay of enzymatic activity. (**A**) Enzymatic assay of wild-type hOGG1 (WT) and hOGG1(K249H) at pH 6.0. The enzymatic assay relies on the detection of enzymatically or chemically driven cleaved substrate with β-elimination, F-β10, or δ-elimination, F-δ10 (referred to as AP-lyase products in the text), by polyacrylamide gel electrophoresis (PAGE) [21], as described in Supplementary Fig. S1. The NaOH-untreated natural conditions [NaOH−: lanes 1, 3, and 5 in panel (A)] reflect the intact AP-lyase activity of hOGG1 alone. In the NaOH-treated conditions [NaOH+: lanes 2, 4, and 6 in panel (A)], the AP-site product can be detected as the AP-lyase products (F-β10 or F-δ10) due to its chemical transformation (Supplementary Fig. S1). A significant increase in the band intensity of the NaOH-treated sample [lane 6 versus lane 5 in panel (A)] indicates the presence of an AP-site derivative. Species present in individual bands are schematically illustrated on the right where F, oG, OH, −CHO, and P denote 5′-fluorescein tag, oxoG, AP-site, aldehyde terminal, and terminal phosphate, respectively. The names of the respective species are shown in parentheses (see Supplementary Fig. S1 for details). (**B**) pH-dependent enzymatic activity of hOGG1(K249H). NaOH-treated samples were analyzed. Weaker enzymatic activity at pH 3.0 would be due to protein denaturation under acidic conditions. The control band [lanes 1 and 2 in panel (A)] was obtained from the substrate DNA that was not processed by the enzyme.

Aliquots of appropriately treated reaction mixtures were subjected to denaturing PAGE with 8 M urea. DNA bands were visualized with Amersham Imager 680 (GE Healthcare Life Sciences, USA) by a fluorescence tag at the 5′-end of the substrate, and analyzed with CS analyzer 4 (ATTO, Japan). For the sake of clarity, positive/negative reversal images are presented (Fig. 2).

### Trapping of the substrate–enzyme covalent adduct

Trapping of the substrate–enzyme covalent adduct is described in Supplementary Materials and Methods.

### Crystallization of the hOGG1 complex

[d(AGCGTCCA(oxoG)GTCTACC)•d(TGGTAGACCTGG ACGC)] was used as a substrate. This DNA sample was dissolved in ultrapure water at 4.0 mM, and added to the hOGG1(K249H) solution at a molar ratio of 1:1.5 [hOGG1(K249H):substrate DNA]. Then, 20 mM Tris–HCl (pH 8.0), 100 mM NaCl, 1.0 mM EDTA, and 5–10 mM dithiothreitol (DTT) were added to the solution of the hOGG1(K249H)–DNA complex to adjust the concentration of the complex at 10.4 mg/ml. They were incubated in a cold room at 4°C for at least 2 h.

Crystallization of the hOGG1(K249H)–substrate DNA complex was performed by a hanging-drop vapor diffusion method using 24-well cell culture plates (Falcon). The crystallization solution consisted of 0.1 M sodium cacodylate buffer (pH 8.0), 0.1–0.2 M MgCl_2_, 20% or 22% (w/v) polyethylene glycol 4000 (PEG4000), and 5% (v/v) glycerol. One microliter of the crystallization solution was mixed with 1.0 μl of the hOGG1(K249H)–substrate DNA complex solution (pre-incubated as described above), on water-repellent-coated cover glasses. The wells containing 200 μl of the crystallization solution as the reservoir solution were covered with the cover glass and sealed with silicone grease. Each plate was stored at 4°C. The derived crystals were used to determine the structure, prior to the enzymatic reaction.

To determine the crystal structures after the reaction in the crystal, the crystals were picked up using cryo-loops and soaked in a reaction solution [acetate buffer pH 4.0, 0.1–0.2 M MgCl_2_, 20% or 22% (w/v) PEG4000, 5% (v/v) glycerol] at 4 or 25°C, for various periods of time (Supplementary Table S2).

Both pre- and post-reaction crystals were picked up with cryo-loops, soaked in a cryoprotectant solution [same as the crystallization (pH 8.0) or reaction (pH 4.0) solution, except that the glycerol concentration was 20% (v/v)], flash-frozen in liquid nitrogen, and stored in it until X-ray intensity data collection.

### X-ray intensity data collection

X-ray diffraction experiments were carried out at the Photon Factory (PF; Tsukuba, Japan), beamlines BL-1A and BL-5A in a remote or fully automated measurement mode, or at the Swiss Light Source (SLS; Villigen-PSI, Switzerland), beamline X06SA in a fully automated measurement mode. X-ray diffraction intensity data collections were performed at ∼-173°C under a cold nitrogen stream to avoid X-ray irradiation damage. Diffraction data collection conditions are listed in Supplementary Table S3.

### Structure determination and refinement

X-ray diffraction data were processed using XDS [22] and aimless [23]. The initial phases were determined by the molecular replacement method using Molrep [24] of CCP4 [25] in which the inactive hOGG1 mutant K249Q–8oxoG DNA complex structure (PDB ID: 1EBM) [13] was used as the initial model. The molecular models were then manually built and corrected using the graphics program Coot [26]. Refmac5 [27] of CCP4 and phenix_refine of Phenix [28] were used for structural refinements; the manual model corrections and the structure refinements were carried out repeatedly. PyMOL (The PyMOL Molecular Graphics System, Version 2.0, Schrödinger, LLC) was used to make figures. The statistics for the crystallographic data and structure refinements are summarized in Supplementary Table S4.

### Kinetic analyses of the enzymatic reaction

Kinetic analyses of hOGG1(K249H) were performed at 15°C in a solution consisting of 50 mM citric acid–Na_2_HPO_4_ buffer (pH series 3.0–8.0), 100 mM NaCl, 5.0 μM DNA duplex F-oG23•C23, and 10 μM hOGG1(K249H). The reaction mixtures were picked up from the bulk reaction solution at 0.5, 5, 10, 20, 30, 40, 50, 60, 120, 240, and 480 min. The picked-up solution was treated as described in the “Assay of enzymatic reaction of hOGG1” section.

The *k*_obs_ value was calculated from the curve fitting based on the following equation (see the details of the derivation of the equation for Supplementary Materials and Methods in Supplementary data).


(1) \begin{eqnarray*}
\left[ {\mathrm{P}} \right] = {{\left[ {\mathrm{S}} \right]}_0}\left[ {1 - {\mathrm{exp}}\left( { - {{k}_{{\mathrm{obs}}}}{t}} \right)} \right] + C ,
\end{eqnarray*}

where [P], [S]_0_, *k*_obs_, *t*, and *C* denote the concentration of the product, the initial concentration of the substrate, the pseudo-first-order rate constant, reaction time, and offset value of the theoretical curve, repectively. The longest times (120, 240, and 480 min) were discarded as the reaction was saturated due to denaturation of the enzyme. The time course of the reaction was recorded four times (*n* = 4), and the *k*_obs_ values and their standard deviations (SDs) are listed in Supplementary Table S5. These values were calculated with the program Excel (Microsoft).

### p*K*_a_ analysis of the active residue

If protonation of an active residue is the prerequisite of enzyme activation, the enzymatic activity increases with acidity. As this activation is proportional to the protonation ratio of the catalytic residue, the pH–activity (*k*_obs_) profile is expected to be sigmoidal. On the other hand, the theoretical value of the pseudo-first-order rate constant, *k*_calc_, can be expressed by using a fraction of the protonated enzyme, *f*(E•H^+^), and a conversion factor, *k*′, between *f*(E•H^+^) and *k*_calc_ (see the details of the derivation of the equation for Supplementary Materials and Methods in Supplementary data).


(2) \begin{eqnarray*}
{{k}_{{\mathrm{calc}}}} = {k^{\prime}}f\left( {{\mathrm{E \cdot }}{{{\mathrm{H}}}^{\mathrm{ + }}}} \right) = \frac{{{k^{\prime}}}}{{1 + {{{10}}^{\left( {{\mathrm{pH}} - {\mathrm{p}}{{K}_{\rm a}}} \right)}}}} .
\end{eqnarray*}

The p*K*_a_ value of the catalytic residue was determined by the least square fitting between the *k*_obs_ values and the respective *k*_calc_ values of the corresponding pH with the program Igor Pro 9 (WaveMetrics).

### ESI-MS analysis of the enzymatic reaction product

The solution-phase enzymatic reaction was performed for 30 min at 15°C and 37°C in a solution consisting of 50 mM citric acid–Na_2_HPO_4_ buffer (pH 5.0), 100 mM NaCl, 20 μM DNA duplex F-oG23•C23, and 80 μM hOGG1(K249H). The reaction mixtures were purified using SepPak Plus C18 cartridges (Waters, Milford, MA, USA). The solution thus derived was subjected to a direct-infusion ESI-MS analysis performed on a Synapt G2-Si HDMS mass spectrometer fitted with an ESI source (Waters) and operated in a negative ESI mode. Data were processed by MassLynx V4.1 (Waters).

### QM/MM calculation

The QM/MM calculation is described in Supplementary Materials and Methods. Other theoretical studies have been reported in the literature [16, 29–37].

## Results and discussion

### Transformation of hOGG1 into a pH-responsive monofunctional enzyme

To obtain a pH-responsive monofunctional mutant of hOGG1, we created a mutant where the catalytic K249 residue was substituted with histidine (H) as explained in the “Introduction” section. We then examined the catalytic activity of the designated hOGG1(K249H) mutant as described elsewhere [21] (Fig. 2); slight activity was detected at pH 6.0 (lane 6 in Fig. 2A). Considering the p*K*_a_ value of the imidazole group in histidine (∼6.0), the activity might be affected by the solution pH, and it was indeed the case; the activity increased at low pH (Fig. 2B). We thus obtained a pH-responsive (activable) mutant exhibiting a sufficient degree of enzymatic activity. This made it possible to “trap” the reactive intermediate directly *in situ* (in the crystal).

To characterize the hOGG1(K249H) mutant in detail, we analyzed its enzymatic activity as either monofunctional (glycosylase activity alone) or bifunctional (glycosylase/AP-lyase activities). Unlike the wild type, no AP-lyase product was detected for the mutant under natural conditions (NaOH-untreated conditions) where the intact AP-lyase activity is detected (lane 5 in Fig. 2A). Upon NaOH treatment, a significant amount of the artificially cleaved AP-lyase products was detected (lane 6 in Fig. 2A; see Supplementary Fig. S1 for details). As NaOH treatment enabled a chemical generation of the AP-lyase products from the apurinic/apyrimidinic site (AP-site), the AP-site was likely generated on the DNA substrate strand by the glycosylase activity of hOGG1(K249H).

**Figure 3.**
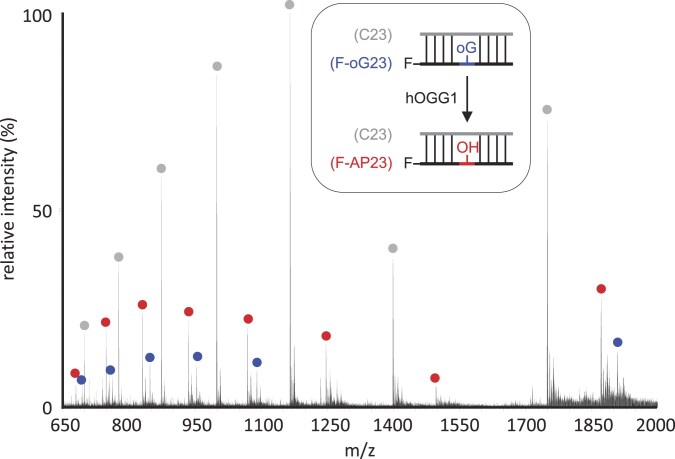
The ESI-mass spectrum of the reaction product of hOGG1(K249H). Blue circle: uncleaved substrate DNA (F-oG23). Red circle: the enzymatically oxoG-base-depleted product with the AP-site (F-AP23). Gray circle: complementary strand to F-oG23 (C23). They were observed as multivalent ions with different *z* (electronic valence). Corresponding *m*/*z* peaks are listed in Supplementary Table S1. The enzymatic reaction products were obtained from the reaction at 37°C. We confirmed that the obtained enzymatic product was the same as that at 15°C (Supplementary Fig. S17).

To prove that the intact enzymatic product of hOGG1(K249H) without any artificial treatment (e.g. NaOH treatment) was indeed the AP-site-containing DNA, we performed an ESI-MS analysis (Fig. 3). The relevant DNA fragment (F-AP23) was detected, jointly with the uncleaved substrate (F-oG23) and their complementary strand (C23) (Fig. 3 and Supplementary Table S1). Thus, hOGG1(K249H) surely did produce an AP-site on the substrate strand, and does possess a canonical glycosylase function, thus yielding an AP-site at the oxoG site as its final product, while at the same time lacking the AP-lyase activity.

We further confirmed the absence of AP-lyase activity in hOGG1(K249H) by NaBH_4_-reductive trapping of the Schiff base intermediate of the AP-lyase reaction (Supplementary Fig. S2) [7, 8, 16]. Indeed, a sodium dodecyl sulfate–polyacrylamide gel electrophoresis (SDS–PAGE) band corresponding to the hOGG1(K249H)–DNA cross-linked adduct containing the reduced Schiff base intermediate moiety was missing (Supplementary Fig. S2). Thus, the hOGG1(K249H) mutant is a monofunctional enzyme with glycosylase activity alone. This is essential for trapping the reaction intermediate state of the enzyme-substrate complex.

### Crystal structure of the hOGG1(K249H)–DNA complex and trapping of the intermediate state in crystal

To reveal the mechanism of action of the hOGG1(K249H) mutant, we determined a three-dimensional (3D) structure of its complex with the substrate DNA, by X-ray crystallography. As the hOGG1(K249H) mutant is almost inactive under basic conditions (pH >7.0), we crystallized the hOGG1(K249H)–DNA complex under weakly basic conditions (pH 8.0). We then soaked the crystals in a reaction solution (pH 4.0), and flash-froze them to arrest the reaction and capture the reaction intermediate directly *in situ* (in the crystal itself).

**Figure 4.**
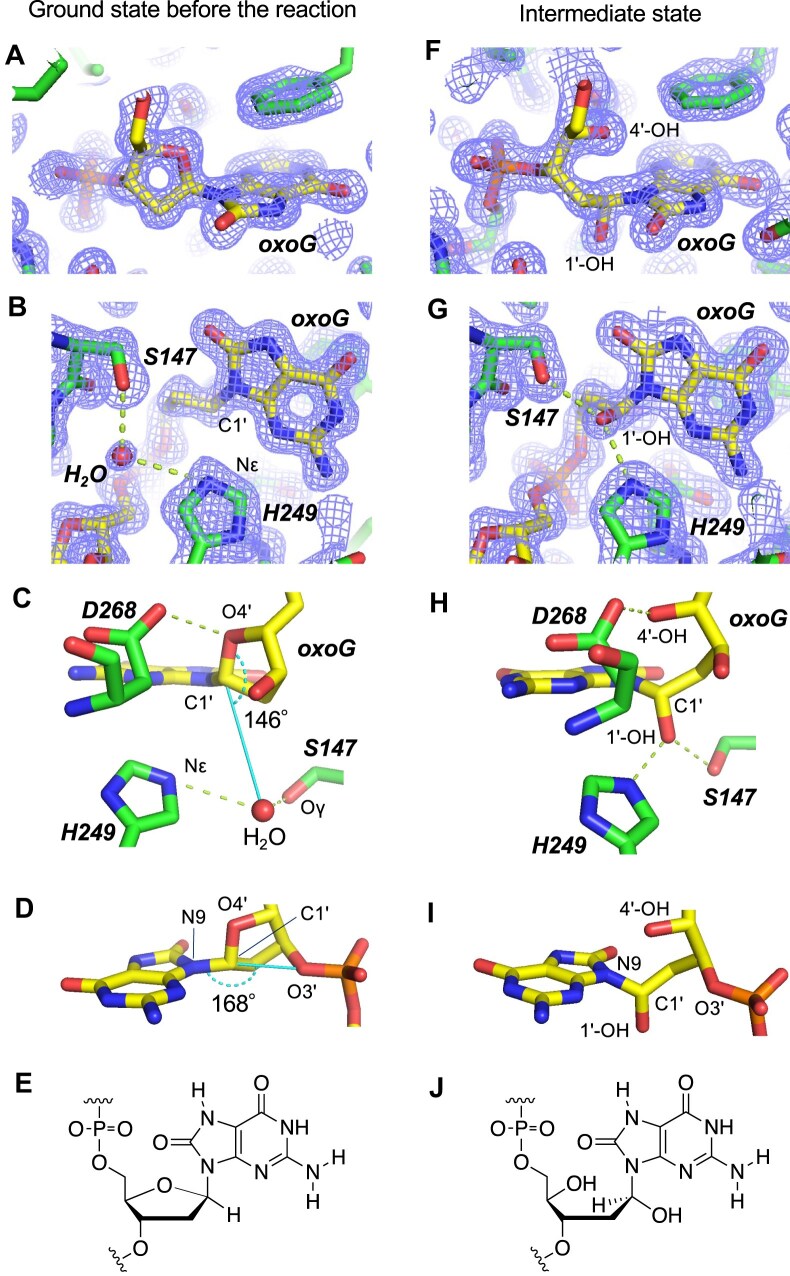
Crystal structure at individual reaction stages. (**A**–**D**) Pre-reaction 3D structure (ground state) of the hOGG1(K249H)–substrate complex. (**A**, **B**) The 2*F*_o_–*F*_c_ maps at 1.5*σ* contour levels at 1.45 Å resolution with the stick models viewed from different directions. The broken lines indicate putative hydrogen bonds. (**C**) Interaction of oxoG with catalytically important residues, and the O4′–C1′–O(H_2_O) angle. (**D**) Conformation of oxoG, and the N9–C1′–O3′ angle. (**E**) Pre-reaction chemical structure of the substrate. (**F**–**I**) Three-dimensional structure of the hemiaminal intermediate obtained in a crystal soaked in a solution of pH 4.0 for 24 h under 4°C, and then flash-frozen at -173°C. (**F**, **G**) The 2*F*_o_–*F*_c_ maps of 1.5*σ* contour level at 1.68 Å resolution with the stick models viewed from different directions. (**H**) Interaction of the hemiaminal intermediate with the catalytically important residues. (**I**) Conformation of the hemiaminal intermediate. (**J**) Chemical structure of the hemiaminal intermediate.

In this way, we obtained a catalytically active hOGG1(K249H) mutant crystallized in a complex with an unreacted substrate, and determined the 3D structure of the pre-reaction ground state of the complex (Fig. 4 and Supplementary Fig. S3). Soaking conditions, statistics of X-ray crystallographic data, and 3D structure features are shown in Supplementary Tables S2–S4. The 3D structure of the complex (Supplementary Fig. S3) was very similar to that determined by Vedine’s group [9, 13]. The oxoG base was flipped out from the DNA duplex and bound to the catalytic site of the enzyme (Supplementary Fig. S3). The H249 residue was located within the catalytic pocket, but the distance between Nϵ of H249 [Nϵ(H249)] and C1′ of oxoG [C1′(oxoG)] was 3.9 Å (Fig. 4B and C). The distance indicates that a direct nucleophilic attack of the Nϵ(H249) atom to the C1′(oxoG) atom is unlikely even if we consider a conformational change of the side chain in H249. On the other hand, a water molecule was found within a hydrogen-bonding distance from Nϵ(H249) and Oγ of S147 [Oγ(S147)] (Fig. 4B and C), which may indicate the catalytic role of the water molecule. In the other catalytic residue, D268, one of its carboxy oxygen atoms is close to O4′(oxoG) (Fig. 4C).

Next, we tried to capture the intermediate state of the substrate, by performing the enzymatic reaction directly in the crystal (see Supplementary Table S2 for experimental conditions). Upon 0.5–2.5 h of soaking in an acidic reaction solution at 4°C, the electron density of the oxygen atom of the putative catalytic water molecule was split into two parts in the flash-frozen crystal (Supplementary Fig. S4). One of them is found at its pre-reaction site, and the other between Nϵ(H249) and C1′(oxoG). Beyond 2.5 h of soaking, the electron density no longer changed much. To be sure the reaction did indeed go through, we soaked one of the crystals for 24 h, and determined its 3D structure (Fig. 4F–I). Notably, the derived substrate structure was unprecedented, with the ribose ring of the oxoG residue opened due to hydroxylation at C1′(oxoG) and conversion of the O4′(oxoG) site into a hydroxy group (Fig. 4I and J). Interestingly, the oxoG base remains attached to C1′(oxoG), yielding a hemiaminal structure at C1′(oxoG) (Fig. 4I and J). Considering that an AP-site-containing DNA is the final enzymatic product (Figs 2 and 3), this hemiaminal compound is the reactive intermediate (an AP-site precursor at the oxoG residue).

**Figure 5.**
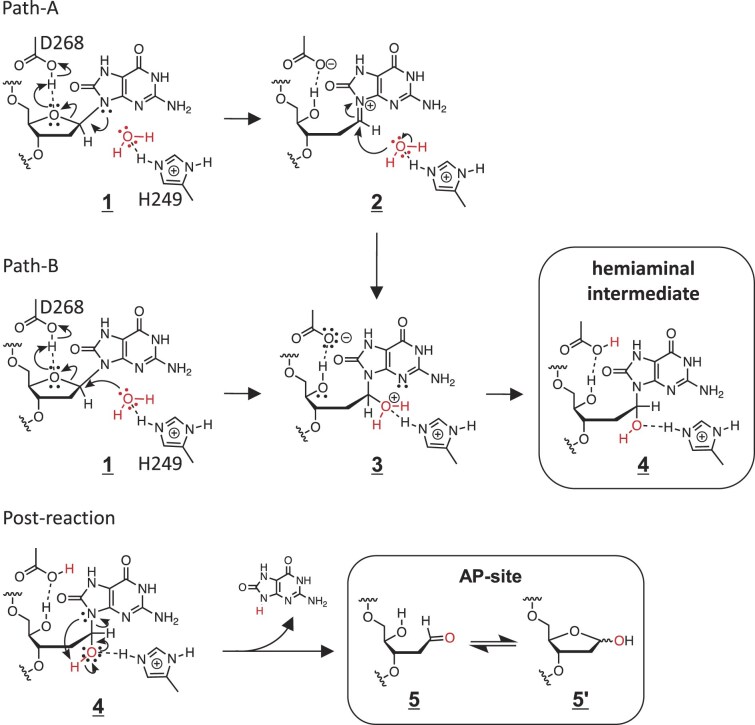
Reaction mechanism, as deduced from experimental data. The respective species are numbered sequentially. In the final step, the aldehyde form **5** would be derived first, and equilibrated with its isomer **5′** according to a general sugar property. The reversibility of the reaction between **1** and **4** due to additional soaking of the crystal at pH 8.0 was explored right after completing the **1**→(**2**)→(**3**)→**4** reaction at pH 4.0; however, the electron density in relevant crystals did not enable concluding the reversibility of the reaction. Note that the one-directional arrows in the reaction scheme do not necessarily indicate irreversibility. The mechanism of the post-reaction was inferred from the electrostatic stabilization mechanism due to hydrogen bond [38, 39]. Alternative reaction mechanisms are discussed in Supplementary Fig. S18.

Combined with data of the final enzymatic product (the AP-site at the oxoG residue), the derived structure of the intermediate state **4** is consistent with a total reaction pathway from substrate **1** to the final product **5**/**5′** (AP-site) (Fig. 5). In this reaction scheme, the protonated D268 residue on its carboxy group functions as a proton donor to O4′(oxoG), thus yielding the ring-opened intermediate. This concept is consistent with the reaction pH (4.0) and the general p*K*_a_ value (∼4.5) of the carboxy group in aspartic acid. D268 thus appears to be the main catalytic residue in the hOGG1(K249H) mutant (Fig. 5).

From a structural point of view, the proton transfer from D268 to O4′(oxoG) is plausible because throughout the reaction, the carboxy oxygen atom of D268 is within a hydrogen-bonding distance from O4′(oxoG) (Fig. 4C and H). In addition, hydroxylation at C1′(oxoG) can be achieved by the nucleophilic attack of the H249-bound water molecule to C1′(oxoG) from the backside of the leaving O4′(oxoG) atom (O4′–C1′–O(water) angle = 146°) (Fig. 4C). In contrast, the attack of the water molecule to C1′(oxoG) from the backside of N9(oxoG) is sterically difficult due to the presence of the O3′ atom in the oxoG residue (N9–C1′–O3′ angle = 168°) (Fig. 4D). This would be the reason why the oxoG base was not eliminated in the intermediate state. The H249 residue seems to act as an anchor for the nucleophilic water molecule. Taken together, we identified the roles of the two key catalytic residues.

We then examined whether the reaction can go on further still, by soaking the crystal for up to 3 weeks as the final product of hOGG1(K249H) in solution is an AP-site-containing DNA (Figs 2A, 3, and 5, and Supplementary Table S2). However, no reaction could be detected (Supplementary Fig. S4B), most probably due to restricted dynamical (hinge) motions in the crystal lattice (Supplementary Fig. S5).

We further considered whether an enzymatic product of the hOGG1(K249H) in solution is AP-site or hemiaminal state. Such discrimination is intrinsically difficult due to the unstable and thus unobservable hemiaminal intermediate within the solution-phase reaction. We recall the facts: (i) the product of the solution-phase hOGG1(K249H) reaction is solely the AP-site and (ii) no obvious chemical role was assigned to the H249 residue during the hemiaminal formation. Based on these observations, we can postulate the following hypothesis: “If the enzymatic reaction of hOGG1(K249H) would be triggered exclusively by the D268 residue, the catalytic activity may be anticipated also for the inactive hOGG1(K249Q) mutant under acidic pH since it contains the D268 residue.” However, the hOGG1(K249Q) mutant did not show catalytic activity under acidic conditions (Supplementary Fig. S6 and Supplementary Table S6). Thus, the mutation of the 249th residue to glutamine (K249Q) makes hOGG1 inactive irrespective of pH, whereas histidine (the K249H mutation) makes hOGG1 active under acidic conditions. These experiments clearly showed the requirement of the H249 residue as a catalytic residue and inferred its chemical role in AP-site formation, such as the electrostatic stabilization mechanism due to hydrogen bond [38, 39] (post-reaction in Fig. 5). These facts let us anticipate that hOGG1(K249H) may have the AP-site forming ability, although more direct evidence is needed for a solid conclusion.

The proposed reaction mechanism of hOGG1(K249H)’s action is to some extent similar to that suggested by Ochsenfeld’s group for wild-type hOGG1; they proposed the ring-opening reaction by the D268 residue as a proton donor to O4′(oxoG) [29]. The 3D structure of the intermediate state obtained in the present study indicated that the D268 residue can be a proton-donating residue in hOGG1(K249H). On the other hand, our enzymatic assay also indicated that this reaction is inefficient under neutral conditions despite activity detected under acidic conditions (Fig. 2B). As wild-type hOGG1 functions at neutral pH, the reaction pathway of hOGG1(K249H) in the present study under acidic conditions cannot be readily anticipated for the physiological mechanism of wild-type hOGG1. In addition to the difference in their optimal pHs, an alternative catalytic pathway triggered due to possibly different protonation states of the principal catalytic residues in the wild type cannot be excluded. On the other hand, a similar pathway to the ring-opened intermediate in this work was proposed for hNEIL1 theoretically [30], and a similar ring-opened “aminal” formation was observed in the crystal structure of the hNEIL1 mutant–DNA complex at pH 6 [40]. This further supports consideration of the ring-opening pathway for wild-type hOGG1; however, the authenticity of the mechanism should be solidly evidenced experimentally.

In summary, the glycosylase reaction intermediate was captured for the first time by using hOGG1(K249H), and the structure indicates that the D268 residue can function as a proton donor, and the H249 residue is likely to maintain the position of the nucleophilic water molecule near the reaction site in hOGG1(K249H). As a result, the oxoG residue in the substrate DNA becomes the ribose ring-opened intermediate with the hemiaminal structure at the C1′ position.

### Identification of the catalytic residue from its p*K*_a_ value

**Figure 6.**
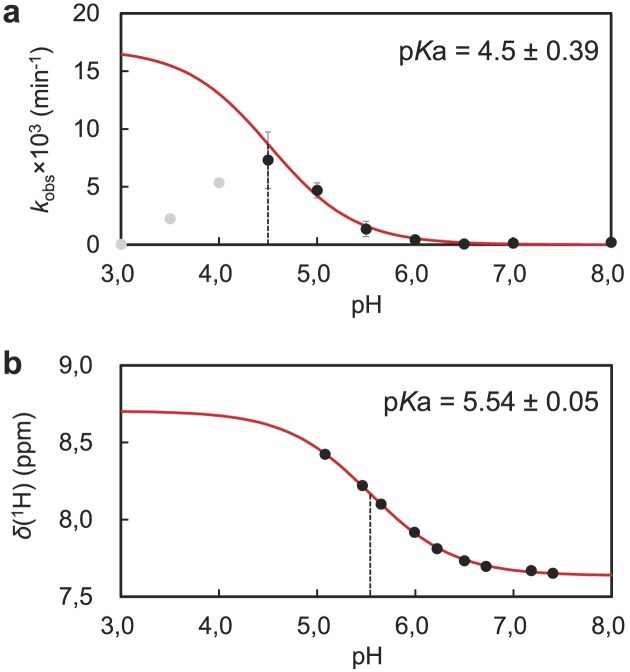
The p*K*_a_ determinations. (**A**) The pseudo-first-order reaction rate constant (*k*_obs_) at each pH with its standard deviation is plotted against pH. The *k*_obs_ values for pH 4.5–8.0 (black circle) were used for the fitting, whereas those for pH 3.0–4.0 (gray circles) were omitted from the fitting since the reactivity was significantly reduced due to a possible denaturation under acidic conditions (Supplementary Figs S7 and S8). It should be noted that the reaction condition satisfied a single turnover condition, as monitored with reaction rates against several enzyme–substrate molar ratios (1/8, 1/4, 1/2, 1, 2, 4, and 10 equivalents) (Supplementary Fig. S19). (**B**) p*K*_a_ of the imidazole ring of the H249 residue determined with NMR spectroscopy. The signal assignment and titration spectra are described in Supplementary Figs S9 and S10, respectively. The theoretical curves based on Equation (2) are depicted as red lines. The resulting p*K*_a_ values are shown in the figure and dashed lines are indicated at the corresponding pH.

As the activity of the hOGG1(K249H) mutant is acidity-driven (Fig. 2B), protonation of the key catalytic residue is expected to trigger the catalysis. To prove that, we determined the p*K*_a_ value of the catalytic residue from a pH–activity plot (Fig. 6A).

As for the enzymatic activity, the pseudo-first-order reaction rate constant (*k*_obs_) was determined at each pH from the time course of the enzymatic reaction (Supplementary Figs S7 and S8, and Supplementary Table S5). As expected, *k*_obs_ values increased as the pH values decreased, which resulted in a sigmoidal/sigmoidal-like pH–activity plot (Fig. 6A). Upon curve fitting, the obtained p*K*_a_ value of the catalytic residue deduced from the enzymatic activity was 4.5 ± 0.39, which correlates well with the p*K*_a_ value of the carboxy group in aspartic acid. This supports the idea that the protonated D268 residue acts as the proton donor (Fig. 5), and is consistent with the reaction pathway deduced from crystallography data alone.

### The p*K*_a_ determination of the H249 residue with NMR spectroscopy

We also determined the p*K*_a_ value of the H249 residue with NMR spectroscopy to exclude the possibility that the p*K*_a_ might arise from that of the H249 residue. To this end, we first performed a mutation-based assignment of Hϵ of the H249 residue (Hϵ(H249)) (Supplementary Fig. S9) and monitored the chemical shift of Hϵ(H249) by pH-titration experiments with ^1^H–^13^C SOFAST-HMQC [41] spectra (Supplementary Fig. S10). The titration curve yielded a p*K*_a_ value of 5.54 ± 0.05 (Fig. 6B), which is within the normal range of the imidazole group in histidine. Importantly, the p*K*_a_ value (5.54) of the H249 residue differs from the enzymatically determined p*K*_a_ value (4.5). Thus, the main catalytic activity was not due to the H249 residue, which again supports the catalytic role of the carboxy group of D268.

### QM/MM calculations and organochemical consideration

To confirm that the hOGG1(K249H) mutant can indeed produce the hemiaminal intermediate, the catalytic pathway linking the unreacted substrate and the hemiaminal intermediate states of the hOGG1(K249H)–DNA complex was modeled by the QM/MM method. The results (based on the crystal structure of relevant states) imply that this reaction pathway does indeed exist. Jointly with an energy diagram, it is shown in Supplementary Figs S11 and S12, and consistent with Path-A in Fig. 5 passing the iminium form **2**. The assumed reaction pathway (Fig. 5) initially deduced from experimental data was thus also predicted theoretically. A detailed description of QM/MM calculations can be found in Supplementary Text and Supplementary Materials and Methods with Supplementary Figs S11–S15.

From an organochemical perspective, we considered why the enzymatic reaction by the hOGG1(K249) mutant proceeds via the hemiaminal intermediate. In principle, the hemiaminal compounds are so unstable [42, 43]. According to organochemical observations, under weakly acidic conditions, the hydroxy group is eliminated to yield enamine, whereas under strongly acidic conditions, the nitrogenous substituent is eliminated to yield aldehyde (Supplementary Fig. S16) [42, 43]. In the ground-state structure of the hOGG1(K249H)–DNA complex, no proton-donating residue can access the N9(oxoG) site (Fig. 4A–C). Considering that the oxoG residue itself is a hemiaminal-like compound with O/N-substituents on C1′(oxoG), the lack of a proton transfer pathway to N9(oxoG) reflects weakly acidic conditions, and the elimination of O4′(oxoG) may thus be favoured. Most likely, this is why the enzymatic reaction by the hOGG1(K249) mutant proceeds via the hemiaminal intermediate, as revealed by the crystal structure.

Once the hemiaminal intermediate is formed, the hydrogen-bond network between (i) the bound hydroxy group at the C1′ position and (ii) the protonated H249 residue, and the adjacent protonated D268 residue may promote strong local acidification around N9(oxoG). This results in the elimination of the N-ligand of the oxoG base, following the empirical organochemical rule (Supplementary Fig. S16). This interpretation suggests that the H249 residue may further act as an indirect acid catalyst for the oxoG-base-elimination step. More broadly speaking, a proton source seems to be indispensable for oxoG elimination, including the reaction mechanism of the wild-type hOGG1. In this sense, it is inferred that the wild-type hOGG1 may take a reaction mechanism shown in Fig. 1D and E [9, 15] or a similar one.

## Conclusion

We obtained an active residue-substituted and monofunctional hOGG1(K249H) mutant, determined the structure of its complex with substrate DNA before and after an enzymatic reaction carried out directly *in situ* (in the crystal), and identified a hemiaminal intermediate as a precursor of the AP-site. Notably, this is the first observation of the reactive intermediate at the glycosylase step operated by hOGG1(K249H). With the aid of QM/MM modeling and organochemical investigations, we identified the likely reaction pathway of hOGG1(K249H) which employs the D268 residue as an acid catalyst. Thus, a reaction mechanism of the glycosylase reaction by hOGG1(K249H) has been proposed.

## Supplementary Material

gkaf718_Supplemental_File

## Data Availability

X-ray structure coordinates and structure factors have been deposited to the Protein Data Bank Japan (www.pdbj.org) and are available under the PDB accession codes: 8XWC, 8XWU, 8XXG, and 8XXK.

## Supplementary data

Supplementary data is available at NAR online.
