# Supplementary material for: Capturing a glycosylase reaction intermediate in DNA repair by freeze-trapping of a pH-responsive hOGG1 mutant
Source: Nucleic Acids Res. 2025 Aug 4;53(14):gkaf718. doi: 10.1093/nar/gkaf718 (PMC12318605; doi:10.1093/nar/gkaf718)
Supplement: gkaf718_Supplemental_File [file gkaf718_supplemental_file.pdf]

Supplementary Data

Capturing a glycosylase reaction intermediate in DNA repair by freeze-trapping of a pH-responsive hOGG1 mutant

Masaki Unno[1,2,*,†], Masayuki Morikawa[3,†], Vladimir Sychrovsky[4], Masataka Koga[1], Nozomi Minowa[1], Saki Komuro[1], Mami Shimizu[1], Mariko Fukuta[3], Fuuka Tsuyuguchi[3], Haruka Mano[3], Yusuke Ochi[3], Katsuyuki Nakashima[3], Yasuko Okamoto[3], Tomohide Saio[5], Yoshikazu Hattori[3,5,*], Yoshiyuki Tanaka[3,*]

Yoshiyuki Tanaka, Masaki Unno, or Yoshikazu Hattori
Email: tanakay@ph.bunri-u.ac.jp, masaki.unno.19@vc.ibaraki.ac.jp, or y-hattori@tokushima-u.ac.jp

† Joint Authors.

CONTENTS:

Supplementary Text
Supplementary Materials and Methods
Supplementary Figures
Supplementary Tables
Supplementary References

SUPPLEMENTARY TEXT

Theoretical model of the reaction pathway

The calculated reaction linking the unreacted and hemiaminal states of oxoG in the hOGG1(K249H)-DNA complex includes a sugar-ring opening reaction and C1'(oxoG)-hydroxylation reaction steps. The IS1 intermediate due to the oxoG sugar ring opening (activation Gibbs free energy, E^a = 12.1 kcal/mol) includes a C1'=N9 double bond (1.325 Å) and a deprotonated amino group of the oxoG base by the D268 carboxyl (**Figures S9 and S10**). Upon sugar-ring opening in the IS1 state (reaction Gibbs free energy relative to the reactant R, E^r = 7.3 kcal/mol), the water molecule shifted closer to the C1'(oxoG) atom (C1'-O(w) = 2.8 Å rather than the original 4.7 Å). An energy-stabilized IS2 intermediate (hemiaminal N3-protonated tautomer of oxoG) was obtained upon hydroxylation of the C1'(oxoG) atom (E^r energy relative to the IS1 state / reactant at -16.6 / -9.2 kcal/mol, activation Gibbs free energy E^a at 19.2 kcal/mol). Further energy stabilization (E^r energy relative to IS2 at -10.1 kcal/mol) was calculated upon transferring a proton from N3(oxoG) in the IS2 state to the amino group of oxoG (**Figure S9**). The reaction Gibbs free energy of the calculated product (P) relative to the reactant (R) was -19.4 kcal/mol (**Figure S10**).

SUPPLEMENTARY MATERIALS AND METHODS

Preparation of substrate DNA and hOGG1

DNA oligomers used in X-ray crystallography (i, ii) and enzymatic/ESI-MS analyses (iii, iv) were purchased from Ajinomoto Biopharma Services-Gene Design (Osaka, Japan) and Tsukuba Oligo Service (Ushiku, Japan), respectively. The following sequences were used: (i) oG16: dAGCGTCCA(oxoG)GTCTACC, (ii) C16: dTGGTAGACCTGGACGC, (iii) F-oG23: d(F)CATCGTTATT(oxoG)ATGACCTGGTGG, (iv) C23: dCCACCAGGTCATCAATAACGATG; oxoG and F denote a residue with the 7,8-dihydro-8-oxoguanine (oxoG) base and a fluorescein (6-FAM) tag at the 5' end, respectively.

The truncated mutant of hOGG1 including the 12th to 327th residues, hOGG1(12–327), was cloned into pET47b vector (Novagen) fused with His-tag, GB1-tag which significantly increases the solubility, and HRV3C protease recognition sequence for cleaving the tags. K249H mutant of hOGG1 was constructed by site-directed mutagenesis PCR based on the PrimeSTAR Mutagenesis Basal Kit protocol (Takara Bio). Recombinant hOGG1 was over-expressed in Rosetta(DE3) or BL21(DE3) strain of *E. coli*. The cells were grown at 37 ºC in M9 minimal medium containing 1 g/L NH_4Cl and 4 g/L D-glucose. For ^{13}C-labeled samples, 1 g/L NH_4Cl and 2 g/L $^{13}C6$-D-glucose were used. Protein expression was induced by 0.5 mM IPTG at OD600: ~ 0.6, followed by a cultivation at 15 ºC overnight. The cells were harvested and resuspended in a lysis buffer of 50 mM sodium phosphate pH 8.0, 300 mM NaCl, 10 mM imidazole, and 10 mM 2-mercaptoethanol (2-Me). The suspended cells were lysed using an ultrasonic homogenizer and centrifuged at 22,000 rpm for 30 min. The protein in the supernatant was purified using Ni-NTA resin (Thermo Fisher Scientific), and HRV3C protease was added to eliminate the tags. After the digestion, the solution was purified using Superdex 75 preparative grade column (Cytiva) equilibrated with 50 mM sodium phosphate (pH 8.0), 300 mM NaCl, and 1 mM ethylenediaminetetraacetic acid (EDTA).

For hOGG1(K249H) used for crystallization, the above described purification procedure was slightly modified with additional chromatographic operations. All purifications were performed in a chromatographic chamber at 4 °C, using an ÄKTA Prime Plus (GE Healthcare) chromatography system. Upon His-tag affinity chromatography purification using HiTrap Q HP 5 mL (Cytiva), the His-tag was cleaved with HRV3C protease, and the enzyme was further purified in two

concatenated columns (HiTrap Q HP 5 mL [top] and Histrap HP 5 mL [bottom] (Cytiva)). Elution was performed with a solution of 5.0 mM NaH_2PO_4 (pH 8.0 by NaOH), 15 mM NaCl, 40 mM imidazole and 0.5 mM 2-Me, and the hOGG1(K249H)-rich fractions were collected. The sample was concentrated on a centrifugal ultrafiltration filter (Amicon Ultra 10k, MERCK) and purified by gel filtration chromatography as described above. The resulting protein solution showing a UV peak was collected and concentrated again by centrifugal ultrafiltration (Amicon Ultra 4K filter, MERCK). Sample concentration was measured using a UV-Vis absorption spectrophotometer (V-650 UV-Vis Spectrophotometer, JASCO), the sample was aliquoted to microtubes, flash-frozen in liquid nitrogen and stored at -80°C until crystallization.

Trapping of the substrate-enzyme covalent adduct

The enzymatic reaction was performed for 15 min at 37 °C in a solution containing 50 mM citric acid-Na_2HPO_4 buffer pH 5.0, 100 mM NaCl, 20 µM DNA duplex F-oG23•C23 and 40 µM hOGG1(WT or K249H). The reaction mixture (10 µL) was mixed with 10 µL of the reducing solution (1 mM $NaBH_4$, 200 mM citric acid-Na_2HPO_4 buffer pH 7.5), and the mixture was incubated for 1, 5 or 15 min at 37°C as described elsewhere(1-3). Aliquots of thus treated reaction mixtures were subjected to SDS-PAGE. The gel was stained with Coomassie Brilliant Blue (CBB) and subsequently destained. The stained proteins were visualized with WSE-5400 Printgraph Classic (ATTO, Japan).

Derivation of the theoretical equation of the enzymatic reaction

The pseudo-first-order reaction rate constant k_{obs} for each condition was determined by curve fitting. Theoretical equation (Eq. S2) of the equilibrium concentration [P] of the reaction product (cleaved DNA) against time (t) was derived from Eq. S1 (kinetics of the substrate's decay).

$$[S] = [S]_0 \cdot \exp(-k_{obs}t) \qquad \text{Eq. S1}$$

$$[P] = [S]_0 - [S] = [S]_0 - [S]_0 \cdot \exp(-k_{obs}t) = [S]_0[1-\exp(-k_{obs}\,t)] \qquad \text{Eq. S2}$$

where [S] and $[S]_0$ denote the equilibrium concentration of the substrate (non-processed DNA) and the initial concentration of the substrate DNA, respectively.

However, the actual enzymatic reaction exhibited a number of unassumed features. This included a non-enzymatic generation of the cleaved product at the oxoG site, resulting in elevated values of [P] at all time points. To compensate for that, an offset (C) had to be added to Eq. S2:

$$[P] = [S]_0[1-\exp(-k_{obs}\,t)] + C \qquad \text{Eq. S3, } \mathit{(1)}$$

This Eq. S3 (equivalent to the equation *(1)* in the main text) was used for calculating the k_{obs} using curve fitting.

pK_a analysis of the active residue

If protonation of an active residue is the prerequisite of enzyme activation, the enzymatic activity increases with acidity. This activation is proportional to the protonation ratio of the active residue, and the protonation reaction is governed by a reversible equilibrium system. In this case, the resulting pH-activity profile is expected to be sigmoidal. Based on this assumption, the pK_a value of the catalytic residue can be formulated with a pseudo-first-order rate constant. To this end, we postulated that protonated hOGG1(K249H), $E \cdot H^+$, is the active form of the enzyme where E

denotes enzyme. In this case, the reaction rate d[P]/dt should be proportional to [E•H$^+$] (concentration of E•H$^+$) and k_{calc} (theoretical pseudo-first-order rate constant).

$$d[P]/dt \propto k_{\mathrm{calc}} \propto [E\bullet H^+] \qquad \text{Eq. S4}$$

[E•H$^+$] is governed by the solution equilibrium:

$$E + H^+ \rightleftarrows E\bullet H^+ \qquad \text{Eq. S5}$$

The fraction of E•H$^+$, denoted as f(E•H$^+$), can be expressed in terms of pK_a of Eq. S5, as follows:

$$f(E\bullet H^+) = 1/(1+10^{(\mathrm{pH}-\mathrm{p}K\mathrm{a})}) \qquad \text{Eq. S6}$$

Therefore, the theoretical pseudo-first-order rate constant, k_{calc}, can be expressed as follows:

$$k_{\mathrm{calc}} = k'\ f(E\bullet H^+) = k'/(1+10^{(\mathrm{pH}-\mathrm{p}K\mathrm{a})}) \qquad \text{Eq. S7, } (2)$$

where k' denotes a conversion factor between f(E•H$^+$) and k_{calc}. Eq. S7 is equivalent to the equation *(2)* in the main text. For curve fitting, the following value, T, was minimized in the least-squares calculation:

$$T = \Sigma(k_{\mathrm{obs}} - k_{\mathrm{calc}})^2/SD^2 \qquad \text{Eq. S8}$$

where SD denotes the standard deviation of each k_{obs} value. In the least-squares calculation, data at pH 3.0 - 4.0 were omitted as the derived kinetic parameters were unreliable, as explained under Table S5. The pK_a value was calculated with the program Igor Pro 9 (WaveMetrics).

QM/MM calculation

QM/MM calculations were carried out with the aid of the Jaguar 11.9, Impact and Qsite programs.(4-7) The M06-2X(8) DFT functional with the 6–31G(d,p) basis set(9) and OPLS2004 all-atom force field(10) were used to calculate the QM and MM part, respectively. The QM part (69 atoms) decorated by hydrogen-atom caps within the hOGG1(K249H)-DNA complex included oxoG, H249 and D268 residues, as well as one crystallographically detected water molecule in the vicinity of the oxoG and H249 residues (**Figure S11**). In QM/MM calculations, the geometry of atoms in the substrate DNA except the oxoG residue and all hydrogen atoms was constrained in accord with the X-ray crystallography data.

Calculated Gibbs free energy includes the harmonic vibration correction at T = 298.15 K and P = 101,325 Pa. Structural models included all the atoms revealed by crystal-structure analysis of the hOGG1(K249H)-DNA complex; PDB IDs: 8XWC (unreacted oxoG state) and 8XWU (hemiaminal oxoG state). First, hydrogen atoms were added by the Maestro program.(11) Two initial models derived from the 8XWC structure (the unreacted oxoG state at pH 8.0) involve specific protonation states of the H249 and D268 residues: [D268]$^{-1}$, [H249]^0 and [D268]^0, [H249]^0. Their QM/MM optimization specifically highlighted the effect of D268 protonation on the catalytic core's geometry. The calculated distance between the [D268]$^{-1}$ carboxy oxygen and the O4'(oxoG) oxygen (~3.1 Å) matches the 8XWC geometry (~3.2 Å) (**Figure S11A**) better than the distance of the [D268]^0 carboxy oxygen (~2.6 Å) (**Figure S11C**). Thus, protonated D268 carboxy group H-

bonded with O4’(oxoG) sugar oxygen (much like the activation of the oxoG sugar ring opening) is unlikely to be the unreacted state observed in 8XWC under the basic condition (pH 8.0). The geometry-optimized hemiaminal oxoG state corresponds well with the relevant X-ray structure (**Figure S12**). The calculated distance in the D268 residue between the carboxy oxygen and the O4’(oxoG) atom (2.6 Å) matches the X-ray geometry of the putative H-bond (2.8 Å). The calculated length of the C1’-O(1'-OH) bond (1.4 Å) in the oxoG hemiaminal structure matches 8XWU (1.4 Å). The QM/MM-optimized structural models are thus consistent with the X-ray geometries obtained in the crystal.

Calculation of the reaction pathway linking the unreacted and hemiaminal states of oxoG included a stepwise change of the relevant reaction coordinate (scan calculation involving a constrained reaction coordinate) followed by unconstrained geometry optimization (energy minimization). The calculated energy minima and transition states were verified by a vibrational frequency analysis.

The D268 residue in the 8XWC structure at pH 8.0 (**Figure 4a-d**) can be easily transformed into the protonated form at pH 4.0 that is feasible for proton transfer from its carboxy group to the O4’(oxoG) atom within the sugar ring (**Figure S13A**). However, the crystallographically identified water molecule close to the oxoG residue is relatively far from the C1’(oxoG) atom (4.7 Å) to enable its nucleophilic attack upon C1’(oxoG) hydroxylation (**Figure S11B**). Therefore, geometry-optimized 8XWU structure (**Figure S12**) was chosen as the initial model to ‘prepare’ a reactant in the calculated reaction. A preliminary reactant was calculated upon shortening the C1’-O4’ distance in the optimized 8XWU structure. This resulted in (i) a proton transfer from the O4’ atom (opened sugar ring) to the nearby carboxy oxygen atom of the D268 residue, and (ii) an addition of a hydrogen atom to the hydroxyl group at C1’(oxoG) that in turn yielded an “interfacial” water molecule between oxoG and the H249 residue.

A stepwise elongation of the C1’-O4’ bond (a scan calculation involving a constrained reaction coordinate) was carried out to model the gradual opening of the sugar ring of oxoG (**Figure S9**). The hOGG1(K249H)-DNA complex was QM/MM-optimized in each step of the scan calculation that included a stepwise extension of the C1’-O4’ distance (0.1 Å steps). A provisional sugar-opened oxoG state was then optimized without constraining the C1’-O4’ distance. Then, the sugar ring closure was calculated (a backward scan). The geometry of the calculated final reactant was optimized only after an additional forward (sugar opening)/backward (sugar closing) scan calculation cycle. The reactant R (**Figure S9**) comprises (i) an H-bond between the protonated carboxy oxygen of the D268 residue and the O4’(oxoG) atom (**Figure S13A**), and (ii) an interfacial water molecule between oxoG and the H249 residues (**Figure S13B**). The C1’-O4’ distance in the R reactant was then stepwise elongated to reach the putative transitional state of sugar ring opening that was deduced according to maximal QM/MM energy in previous scan calculations. The geometry of the relevant transitional state (TS1* in **Figure S9**) with a vibration frequency of -308 cm^{-1} was optimized without constraining the C1’-O4’ distance. The C1’-O4’ distance was then further extended towards oxoG with opened sugar, and geometry of the relevant intermediate was optimized as well (IS1 in **Figure S9**). The hemiaminal HN3 tautomer of the oxoG residue (IS2 in **Figure S9**) and the relevant transition state (TS2* in **Figure S9**) with a vibration frequency of -149 cm^{-1} were modeled by scan calculation of the C1’(oxoG) – O(water) reaction coordinate. The ultimate hemiaminal oxoG state (P in **Figure S9**) was calculated upon a transposition of the proton at N3(oxoG) to the amino group of oxoG.

SUPPLEMENTARY FIGURES

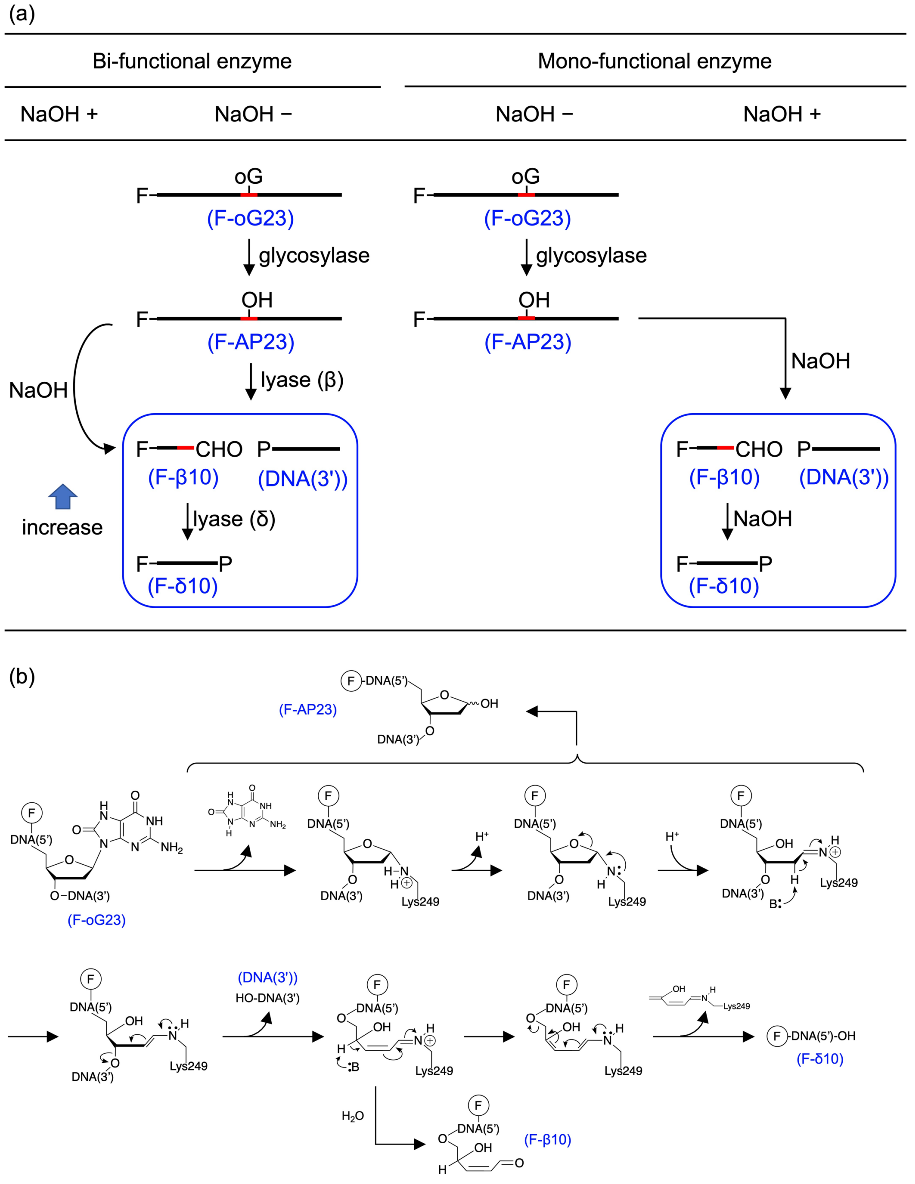

Figure S1. Schematic representation of the enzymatic assay system and enzymatic reaction pathway. (a) The concept of the experiment performed in Figure 2; a schematic representation of the enzymatic reaction pathways for mono-/bi-functional enzymes, and the NaOH-mediated reaction. "NaOH-" denotes a natural enzymatic reaction without any chemical treatment. "NaOH+" denotes an artificial chemical reaction in which the AP-site is converted into the β/δ-elimination products with the NaOH-treatment. (b) Approximate reaction scheme of hOGG1 and its enzymatic products. In both panels (a, b), the names of the key compounds are shown in blue (in parentheses). F-oG23: Fluorescein-labeled 23mer DNA fragment with an oxoG residue. F-AP23: Fluorescein-labeled 23mer DNA fragment with an AP-site. F-β10: Fluorescein-labeled 10mer DNA fragment of the AP-lyase product (β-elimination). F-δ10: Fluorescein-labeled 10mer DNA fragment upon the δ-elimination. DNA(3'): 3' fragment upon the β-elimination.

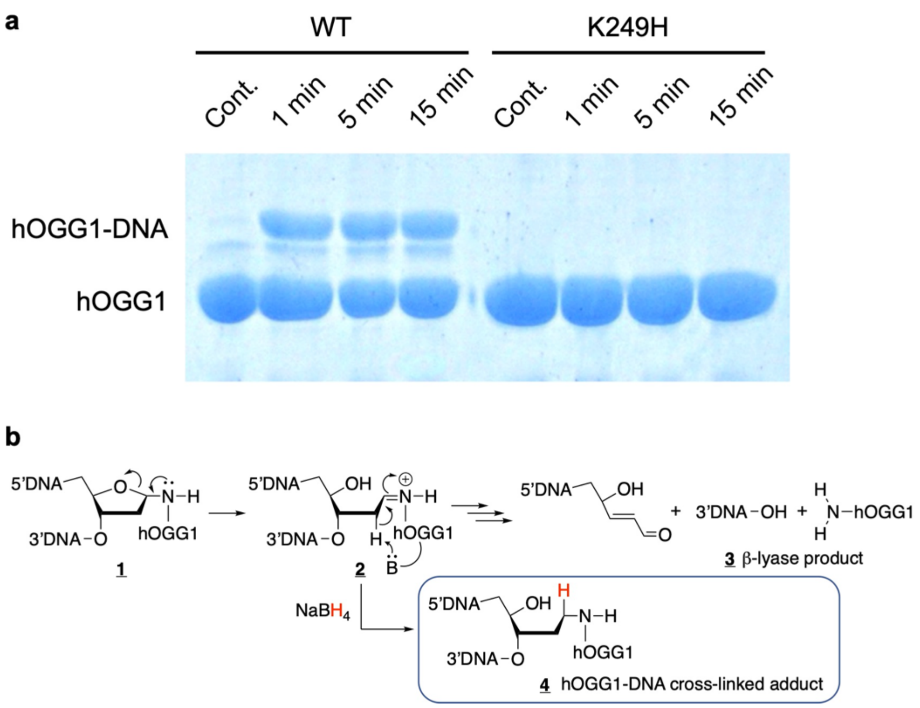

Figure S2. Trapping of a substrate-enzyme covalent adduct upon $NaBH_4$ reduction of a Schiff base intermediate during the AP-lyase reaction.(1-3) If hOGG1(K249H) possesses AP-lyase activity, it should form a Schiff base composed of a transient substrate-enzyme covalent intermediate. This covalent linkage can be irreversibly fixed as a covalent adduct upon the reduction with $NaBH_4$.(1-3) (a) SDS-PAGE of $NaBH_4$-treated hOGG1-substrate DNA complex. Protein bands are visualized with Coomassie Brilliant Blue (CBB). The time course of the reaction is presented for hOGG1(WT) and hOGG1(K249H). The labels "hOGG1" and "hOGG1-DNA" denotes the intact protein (hOGG1(WT) and hOGG1(K249H)) and the hOGG1-DNA cross-linked adduct. The label "Cont." is the lane of the negative control where no $NaBH_4$ treatment was applied to the hOGG1-substrate DNA complex. In hOGG1(WT), the hOGG1-DNA cross-linked adduct was observed, which demonstrated that the experiment was correctly performed. By contrast in hOGG1(K249H), the cross-linked adduct was not observed, which indicates the lack of AP-lyase activity of hOGG1(K249H). (b) The reaction scheme of $NaBH_4$ reduction of the Schiff base site (<u>**2**</u>) in the hOGG1-DNA complex. The resulting hOGG1-DNA cross-linked adduct (<u>**4**</u>) is boxed. The hydrogen atom from $NaBH_4$ is highlighted in red.

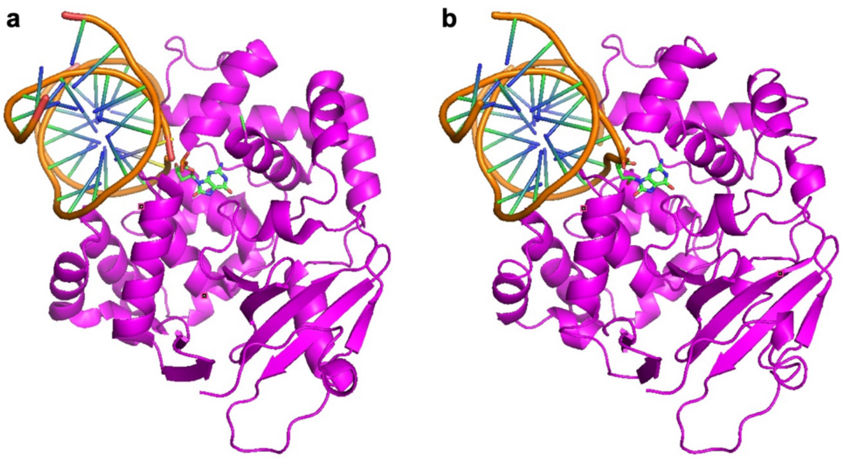

Figure S3. Global 3D structure of the enzyme-DNA complex. (a) Before the reaction. (b) After the reaction. In both panels, protein (hOGG1) is colored in purple. DNA molecule is shown as a tube model (backbone: orange, base: green and blue), and only the oxoG base is shown as a stick model.

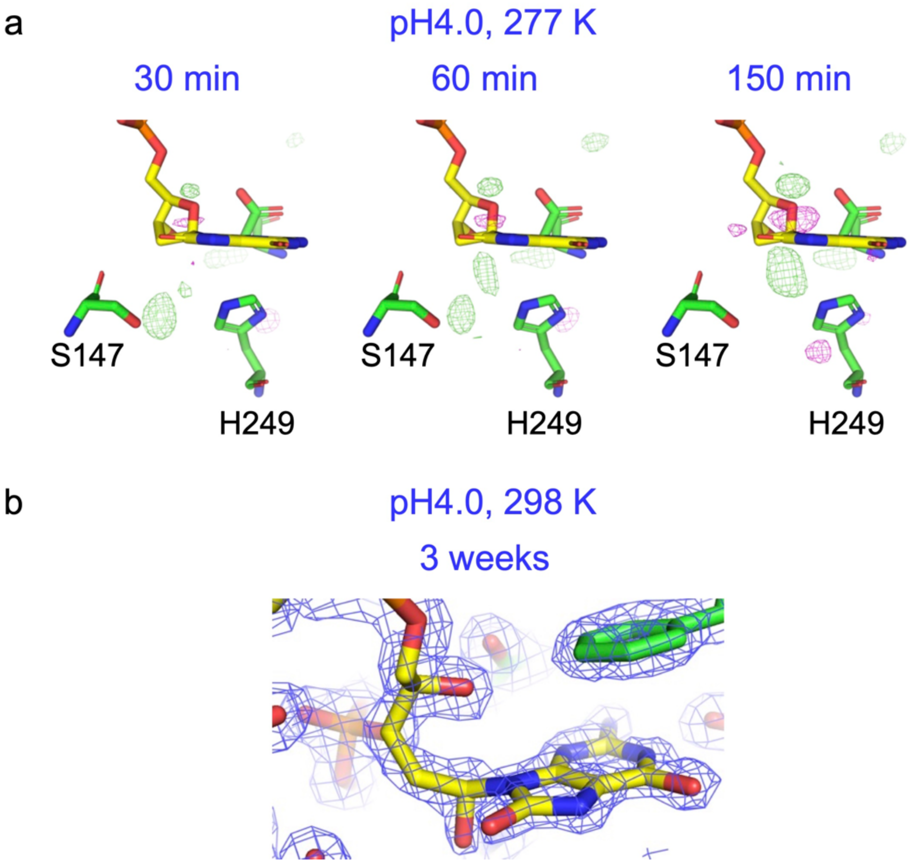

Figure S4. Time course of the electron density. (a) (left) $F_o - F_c$ map calculated using the resting state model at 3.5σ contour level at 1.68 Å resolution after 30 minutes from the reaction start. (middle) The one after 60 minutes from the reaction start. (right) The one after 150 minutes from the reaction start. (b) $2F_o - F_c$ map at 1.5σ contour level at 1.70 Å resolution after three weeks from the reaction start at 298 K. The hemiaminal structure has been kept even after three weeks.

(a)

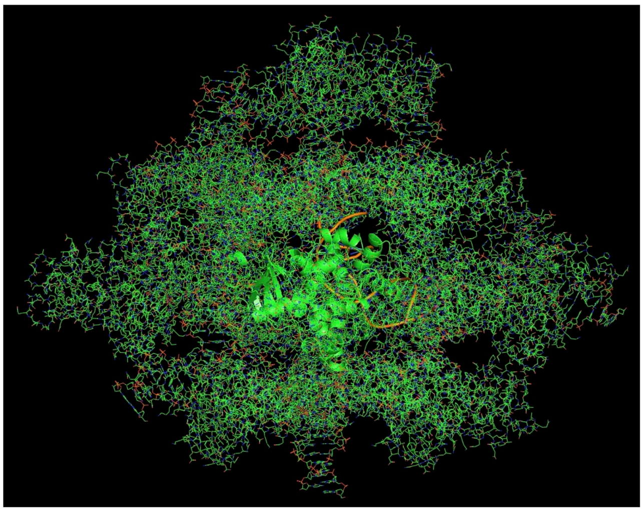

(b) 8XXK (c) 8XXK vs 8XWC (d) 8XXK vs 8XWU

(e) 8XXK vs 1EBM (f) 8XXK vs 1N3C (g) 8XXK vs 1HU0

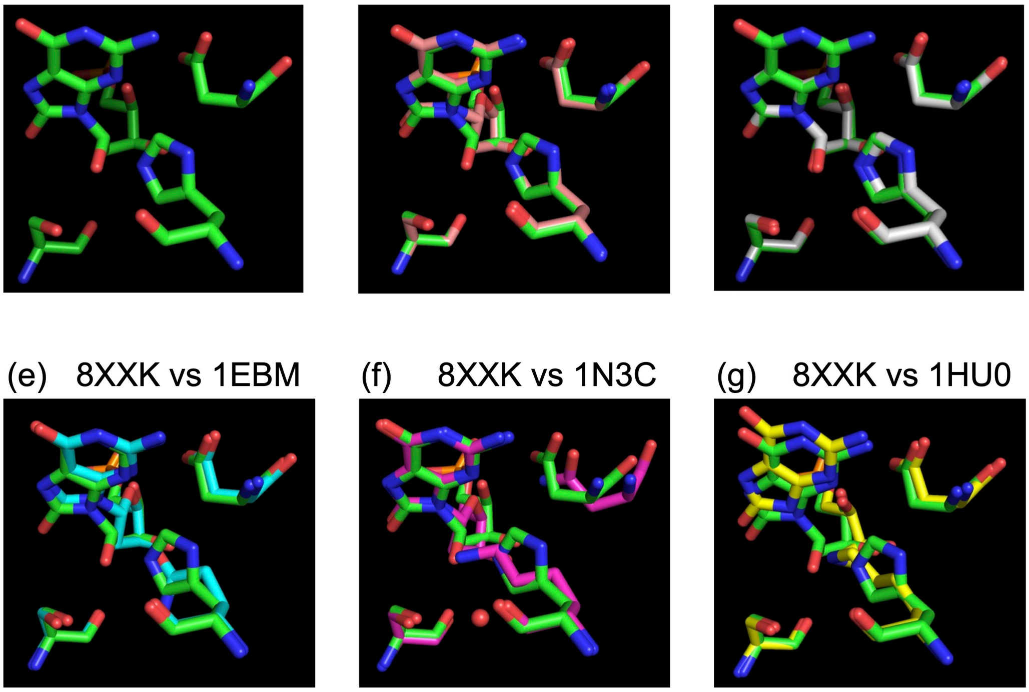

Figure S5. Packing of the hOGG1(K249H)-DNA complexes in crystal (a) and overlay of the catalytic site in the crystal structures (b)-(g), as indicated by color coding: 8XXK green, 8XWC salmon pink, 8XWU grey, 1EBM cyan, 1N3C magenta, and 1HU0 yellow. In (a), one enzyme-DNA complex (ribbon diagram) is closely surrounded by 13 enzyme-DNA complexes (thin wire model). Dense packing of the protein-DNA complexes most probably restricted their dynamical motions (particularly hinge motions) needed to complete the enzymatic reaction that would otherwise proceed in solution. Considerable structural uniformity of the catalytic site (excluding the reacted part of the oxoG residue) was seen in (b)-(g), although group 1 (1EBM, 1N3C, and 1HU0) and group 2 (8XXK, 8XWC, and 8XWU) belong to different crystal lattices. Extreme uniformity of the 8XXK, 8XWC, and 8XWU structures indicated highly constrained hOGG1(K249H) structures due to crystal packing.

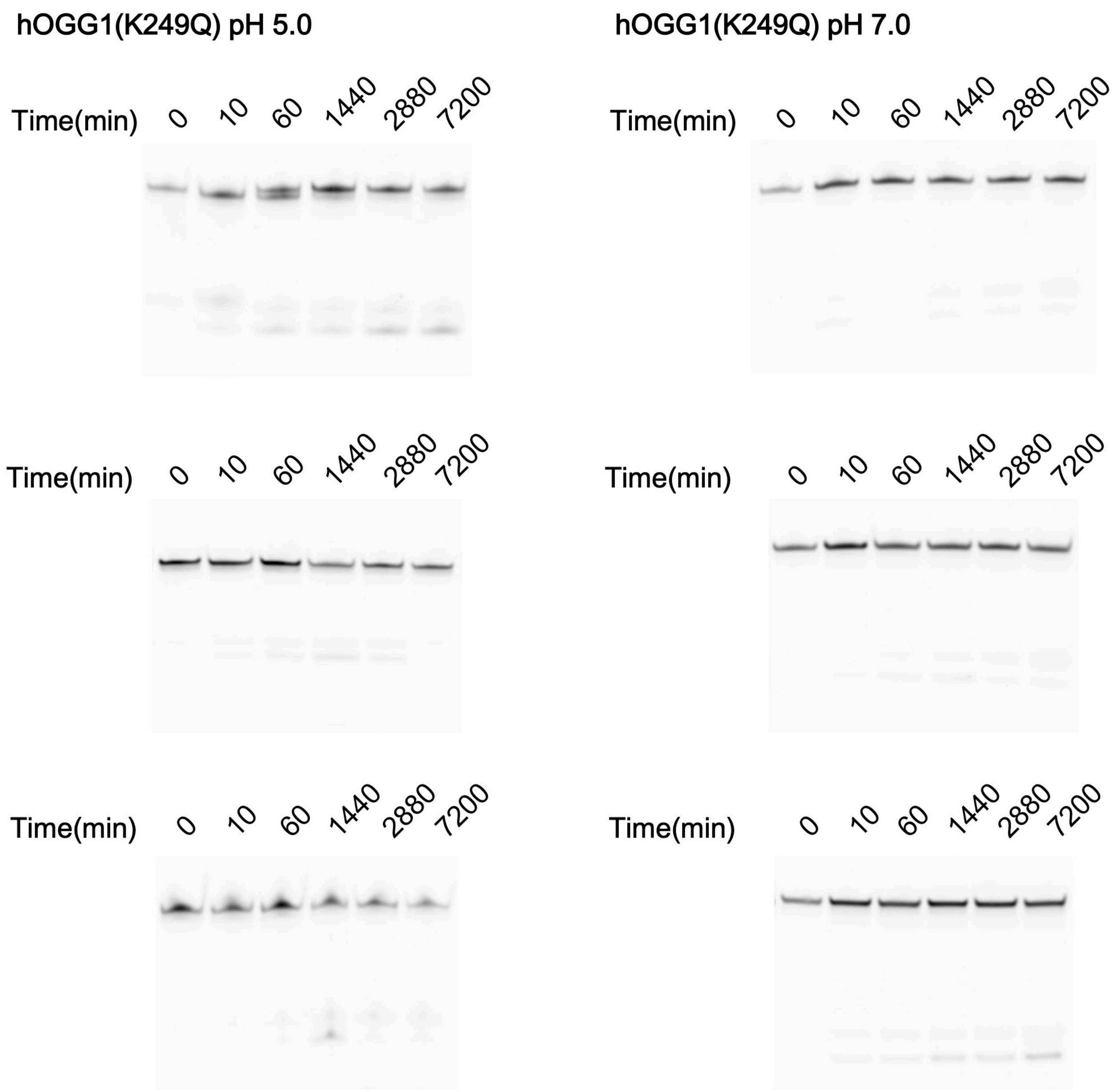

Figure S6. Time course of the enzymatic reactions of hOGG1(K249Q) at pH 5.0 and 7.0. Three independent experiments are displayed for the respective pHs. Even in the case of the maximum-product-observed condition, only a slight amount of the product was produced after five days (7200 minutes).

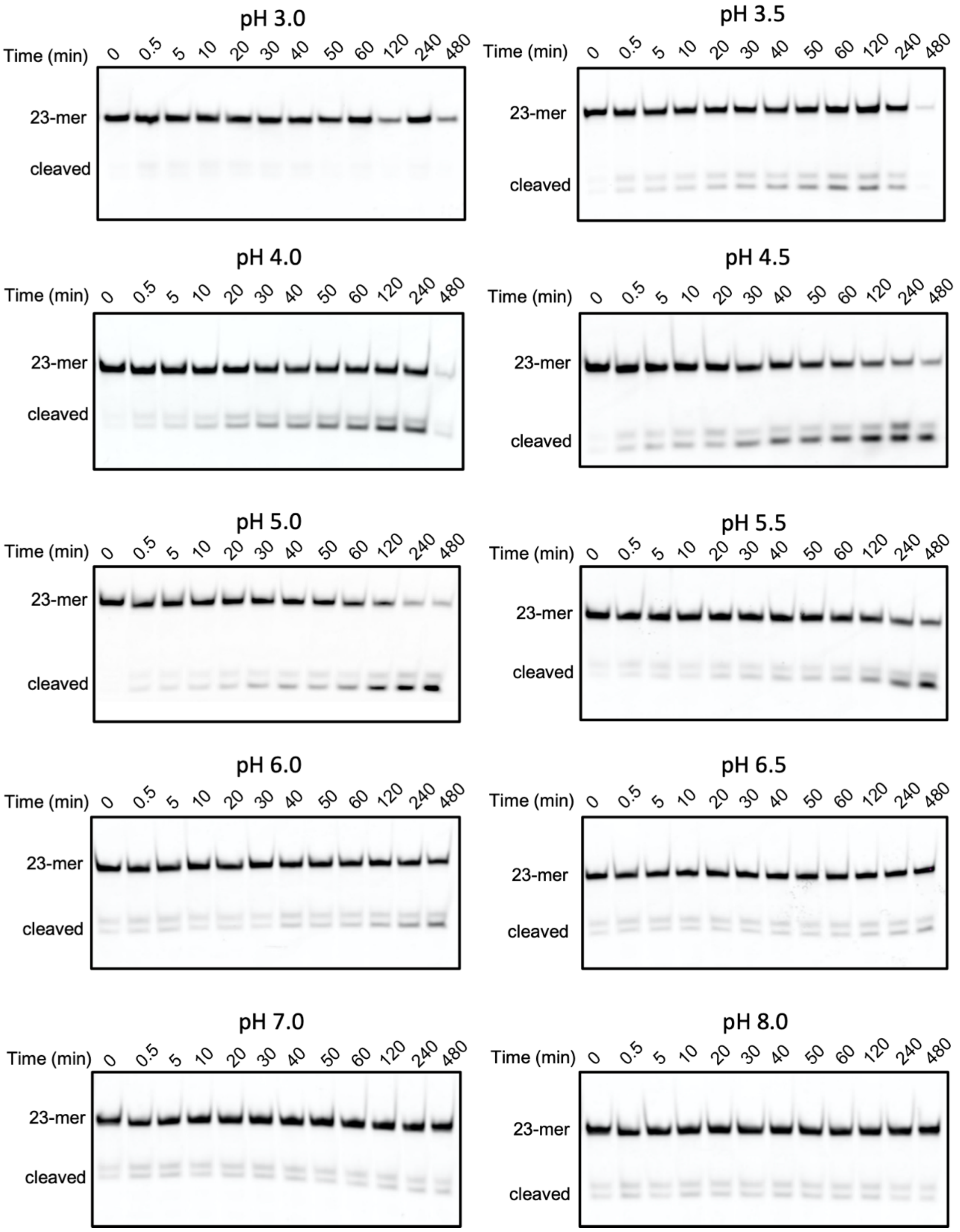

Figure S7. Time course of the enzymatic reaction at various pH values. pH values for the enzymatic reaction are indicated above the gel images. The labels, "23-mer" and "cleaved" denote the uncleaved substrate and the cleaved products, respectively. The timeline of the enzymatic reaction is indicated on top of each gel image.

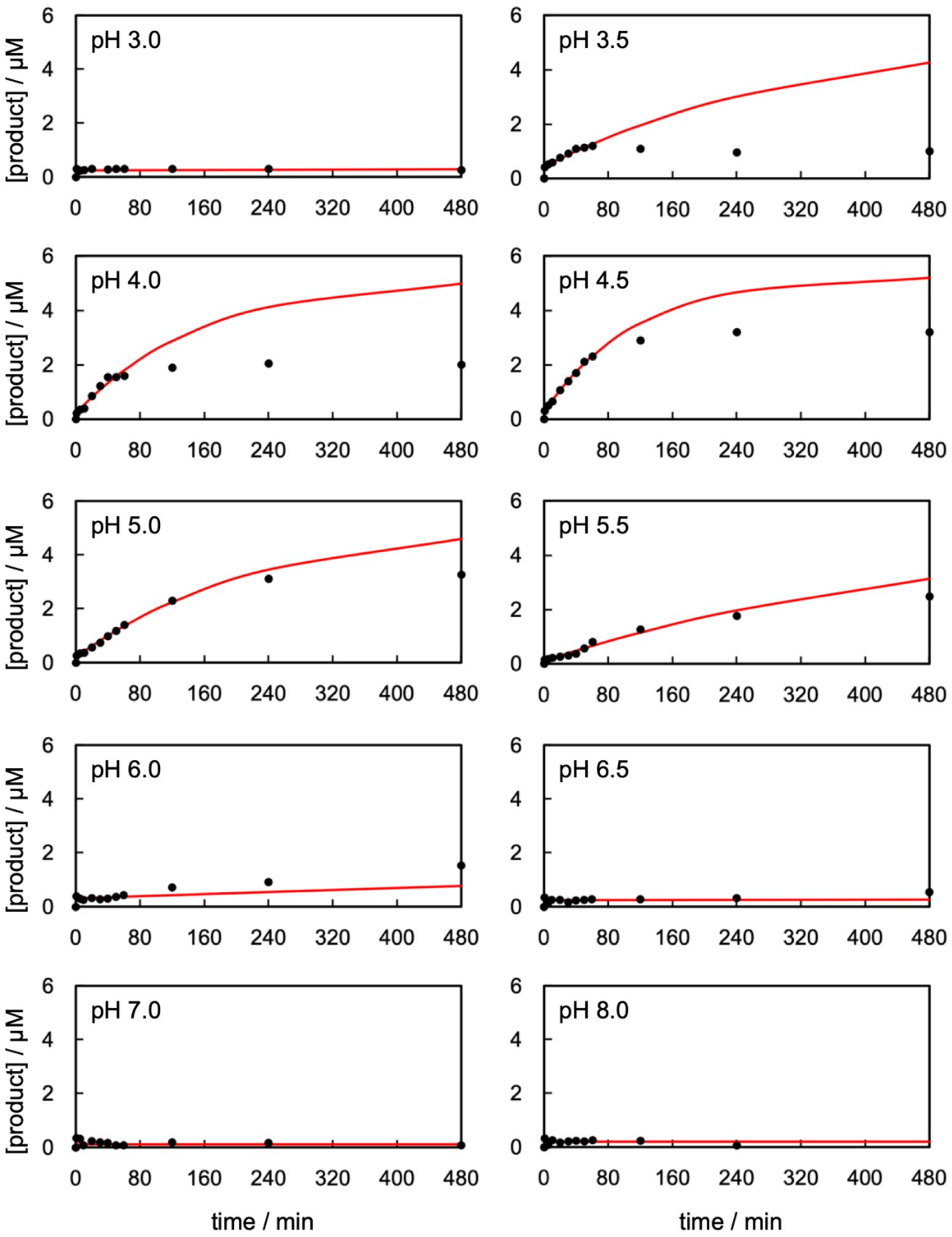

Figure S8. The time course of the glycosylase reaction of hOGG1(K249H). The theoretical curve (red) of the product concentration was calculated using Eq. S3 (equation *(1)*). The curve-fitting against experimental data (black circles) yielded pseudo first-order reaction rate constants (k_{obs}). At each pH, experiments were performed in quadruplicate. A typical series of experiments are shown. After ~2 hours, experimental data started to deviate from the theoretical curve, which suggests a denaturation of the enzyme. They were thus not used to determine the k_{obs} values. The deviation increased as pH decreased.

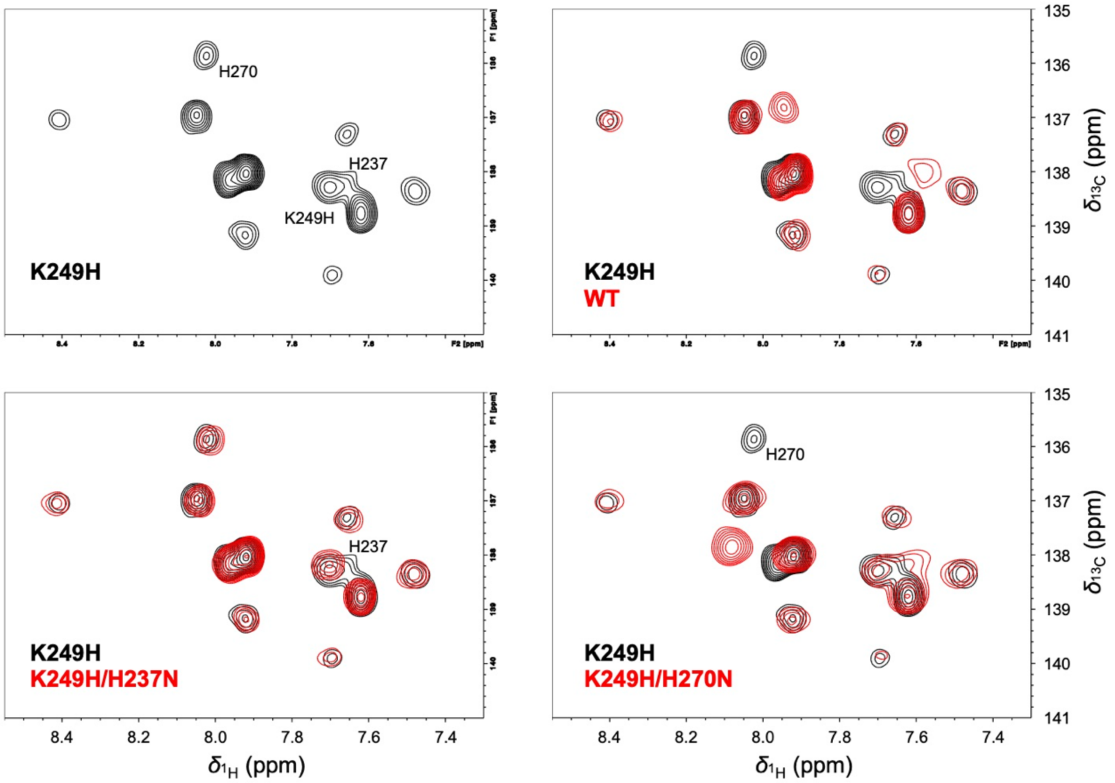

Figure S9. Assignment of the Hε-Cε cross peaks of the H249 residue with ^{1}H-^{13}C SOFAST-HMQC spectra. (Top left) The ^{1}H-^{13}C SOFAST-HMQC spectrum of the hOGG1(K249H) mutant. (Top right) Overlay of the spectra of the hOGG1(K249H) mutant and hOGG1(WT). (Bottom left) Overlay of the spectra of the hOGG1(K249H) mutant and a double mutant, hOGG1(K249H/H237N) in which the H237 residue was additionally mutated to asparagine (N) from hOGG1(K249H). (Bottom right) Overlay of the spectra of the hOGG1(K249H) mutant and double mutant, hOGG1(K249H/H270N) in which the H270 residue was additionally mutated to asparagine (N) from hOGG1(K249H). The ^{13}C-labeled hOGG1 samples at 0.1 mM concentration were dissolved in a solution consisting of 20 mM sodium phosphate buffer pH 6.8, 50 mM NaCl, and 5% ^2H_2O. NMR spectra were measured on Bruker AVANCE-III HD 500 MHz spectrometer at a sample temperature of 25 ºC. SOFAST-HMQC pulse sequence was employed to obtain the histidine Hε-Cε correlation spectrum. The assignment of the Hε-Cε cross peak of H249 was performed with a mutation-based signal assignment using hOGG1(WT), hOGG1(K249H/H237N), and hOGG1(K249H/H270N). In the overlay panels, black spectra are those of hOGG1(K249H), and red ones are those of the counterpart proteins whose names are shown in red. In the top right panel, three non-overlapping Hε-Cε cross peaks were seen. Two of them (H237 and H270) were identified by comparison with the double-mutant spectra (bottom row). The remaining one was identified as H249. All assignments are shown in the top left panel.

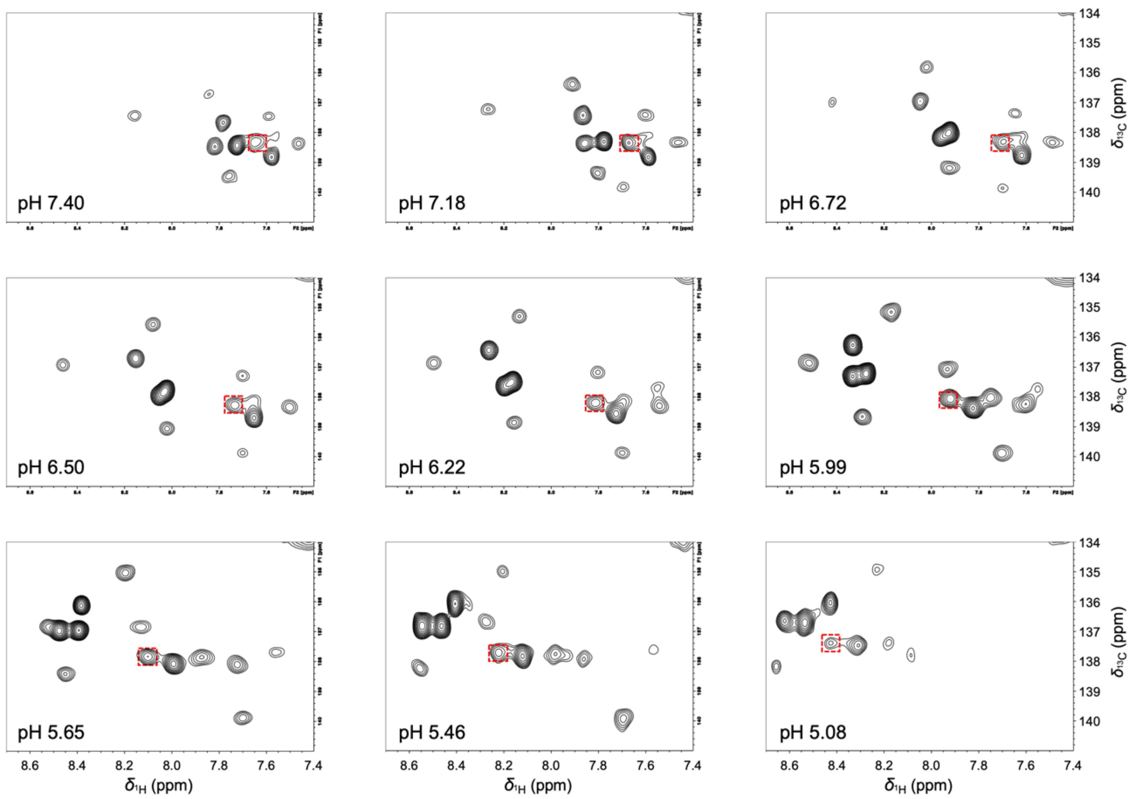

Figure S10. Titration experiments of the H249 residue with ^{1}H-^{13}C SOFAST-HMQC spectra. The Hε-Cε cross peak region of the ^{1}H-^{13}C SOFAST-HMQC spectra is shown at each pH value. The Hε-Cε cross peak of the imidazole ring of the H249 residue is boxed in red. The details of the assignment of the Hε-Cε cross peak of the H249 residue are described in the legend to Figure S9. NMR measurements were performed at an increment of 0.3–0.5 pH units, starting from pH ~6.8 to either (i) acidic pH region adjusted with HCl or (ii) basic pH region adjusted with NaOH, respectively.

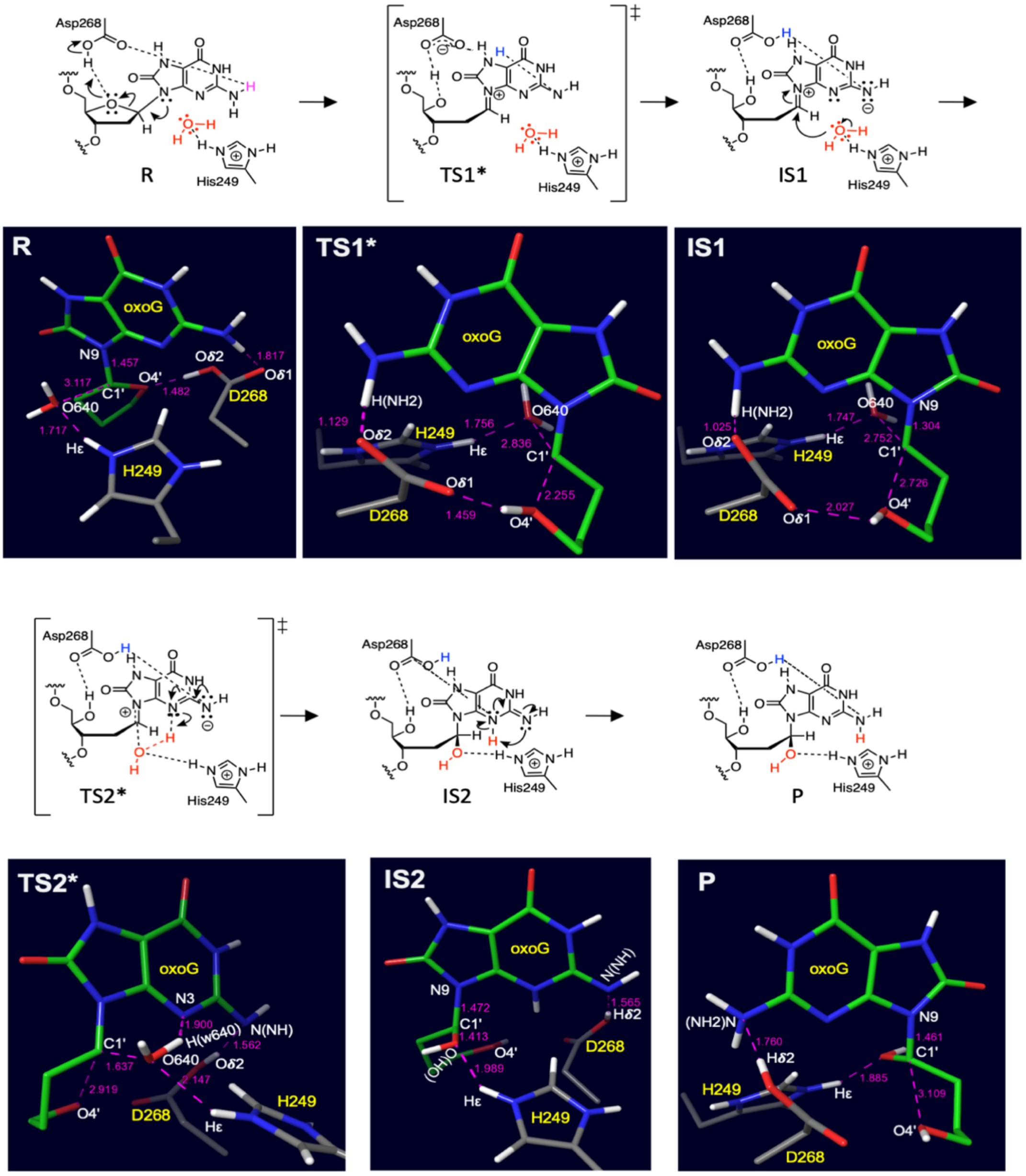

Figure S11. Theoretically modelled reaction of hOGG1(K249H). Chemical schemes and QM/MM-calculated geometries of individual reaction states including the reactant R (unreacted state), transition TS1* state (oxoG sugar ring opening), intermediate IS1 state (sugar-ring-opened oxoG), transition TS2* state (hydroxylation at C1' of oxoG due to the attack of the O atom [water W640] to the C1'atom of oxoG), intermediate IS2 state (a tautomer of hemiaminal oxoG), and the reaction product P (a hemiaminal oxoG intermediate corresponding to the 8XWU crystal structure). Distances between the labelled atoms are shown in Ångströms. Calculated Gibbs free reaction and activation energy values are shown in Figure S10.

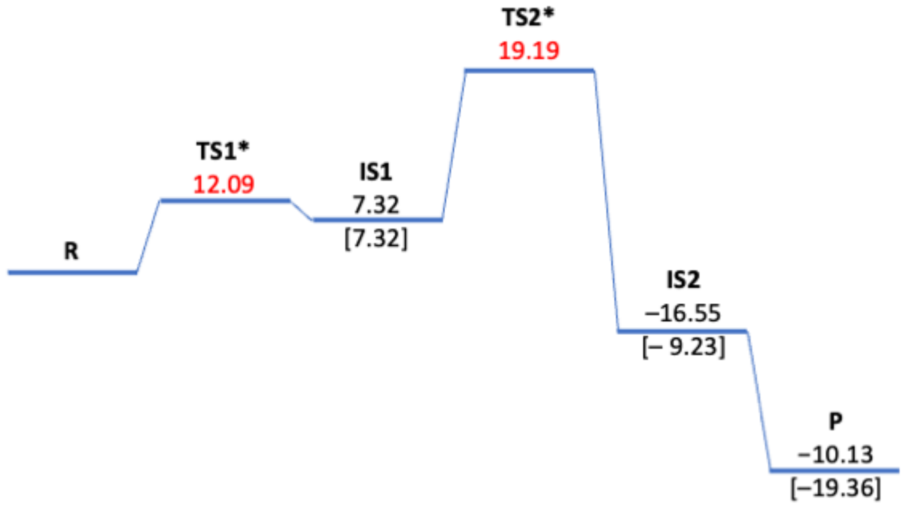

Figure S12. Calculated Gibbs free energy (kcal/mol) of the individual reaction states of hOGG1(K249H). The activation energy of the transition states, TS1* (opening of the oxoG sugar ring) and TS2* (hydroxylation at C1' carbon of oxoG), and reaction energy of the intermediate states IS1 (sugar-ring-openned oxoG), IS2 (hydroxylated oxoG at C1' carbon) and the reaction product P (hemiaminal oxoG) are relative to the Gibbs free energy of the preceding intermediate state. The Gibbs free energy of the intermediate states relative to the reactant R are shown in brackets. The chemical structures and QM/MM-calculated geometries are shown in Figure S9.

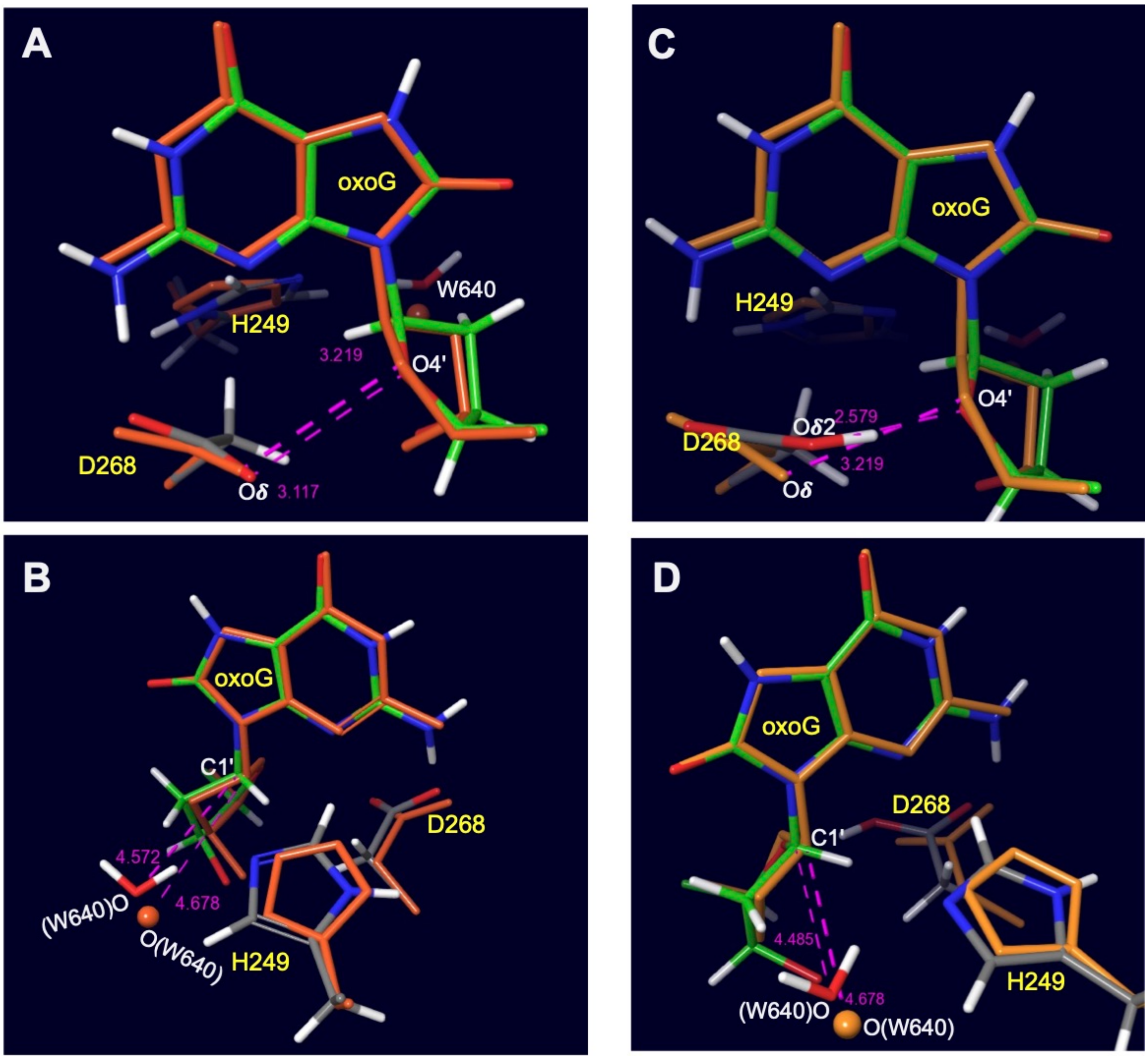

Figure S13. Overlay of experimental 8XWC (orange) and QM/MM-calculated (colour-coded atoms) structures of the catalytic core including the unreacted oxoG residue. The geometry-optimized 8XWC structures of the hOGG1(K249H)-DNA complex including the $[D268]^{-1}$ and $[H249]^{0}$ residues (A, B), and the $[D268]^{0}$ and $[H249]^{0}$ residues (C, D). The oxygen atom of the W640 water molecule (as revealed in the 8XWC crystal structure) is indicated by an orange sphere. Distances between labelled atoms are shown in Ångströms.

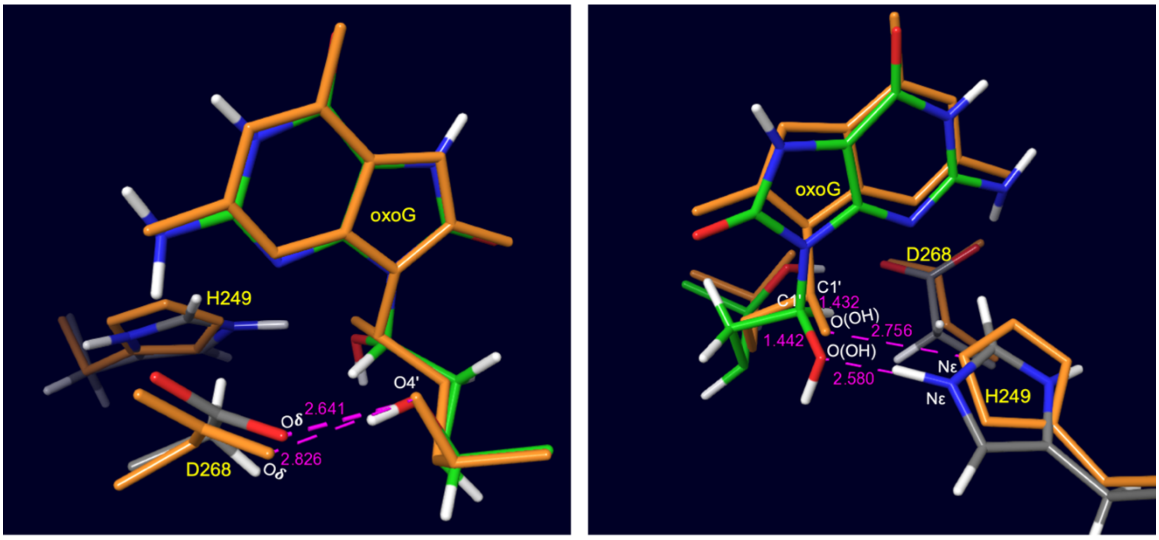

Figure S14. Overlay of experimental 8XWU (orange) and QM/MM-calculated (colour-coded atoms) structures of the catalytic core including the hemiaminal oxoG residue. The QM/MM-optimized 8XWU structure of hOGG1(K249H)-DNA complex includes the $[D268]^{-}$ and $[H249]^{+1}$ residues. Distances between labelled atoms are shown in Ångströms.

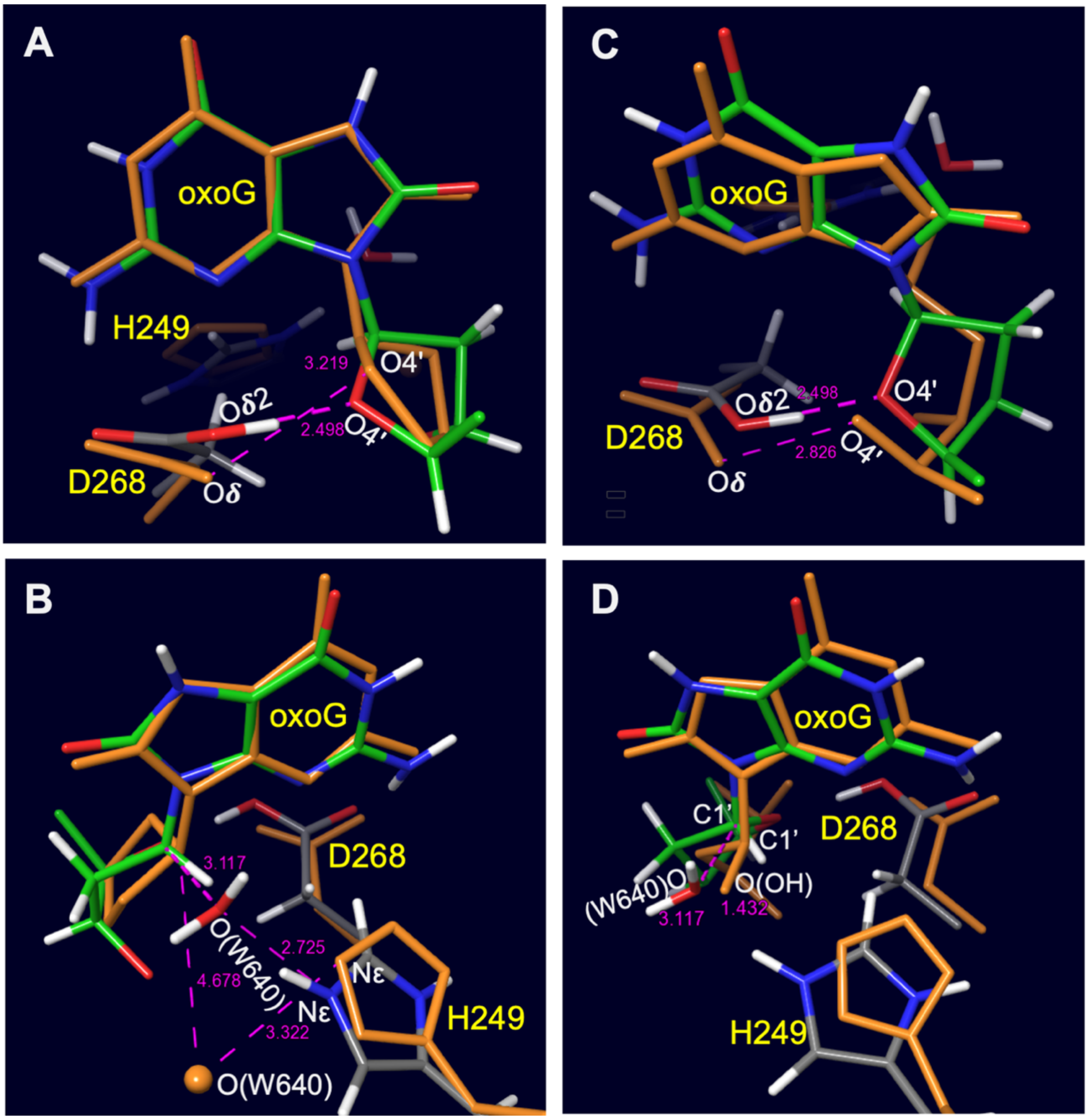

Figure S15. Overlay of experimental (orange) and QM/MM-calculated (colour-coded atoms) structures of the catalytic core. The 8XWC (unreacted oxoG) and geometry-optimized reactant R (A, B). 8XWU (hemiaminal oxoG) and geometry-optimized reactant R (C, D). The oxygen atom of the water molecule W640 (as revealed in the 8XWC crystal structure) is indicated by an orange sphere. Distances between labelled atoms are shown in Ångströms.

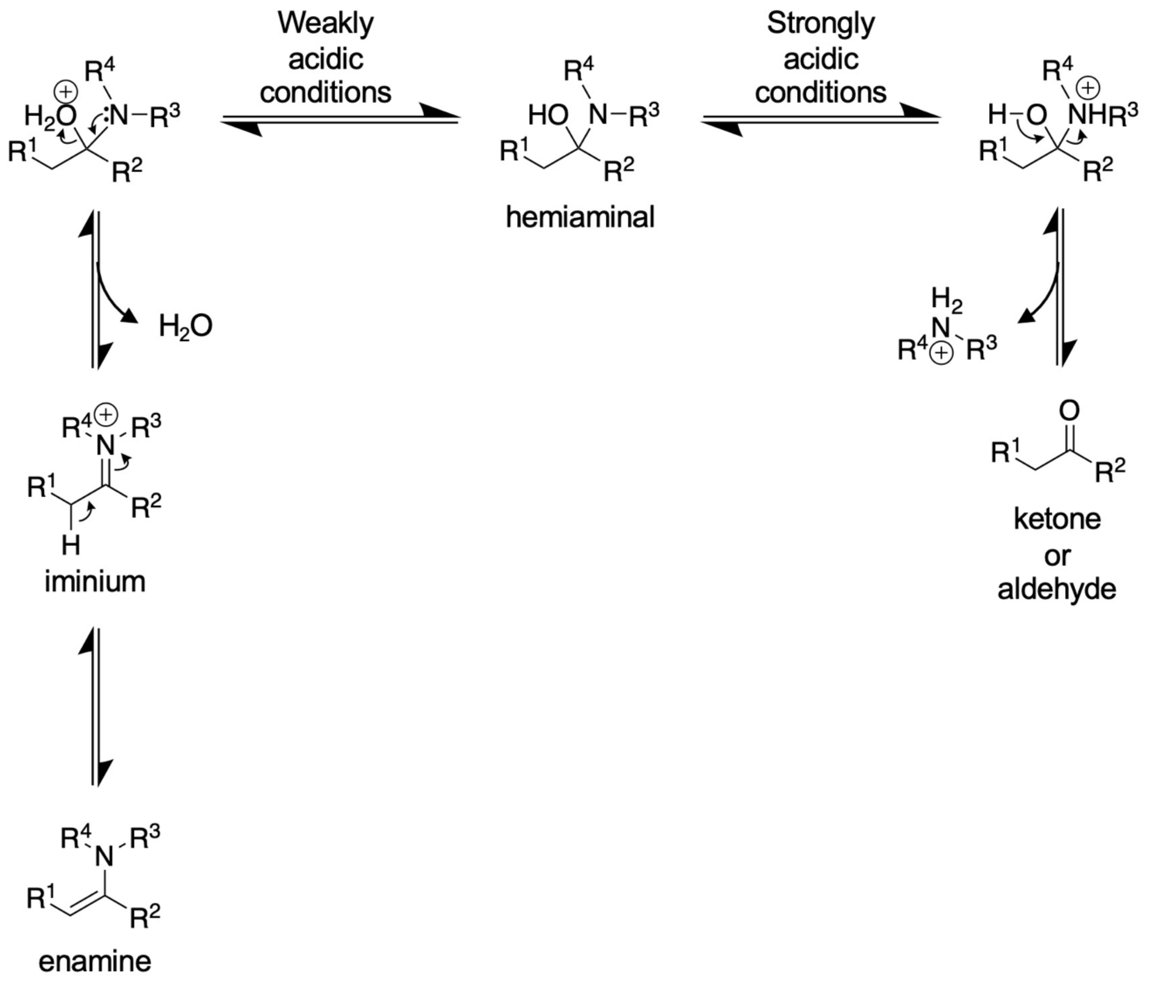

Figure S16. Empirical propensity of the reactivity of hemiaminal compounds. The hemiaminal compound is depicted in the center. The reaction under weakly acidic conditions is shown on the left (enamine is the final product). The reaction under strongly acidic conditions is shown on the right (ketone or aldehyde is the final product).

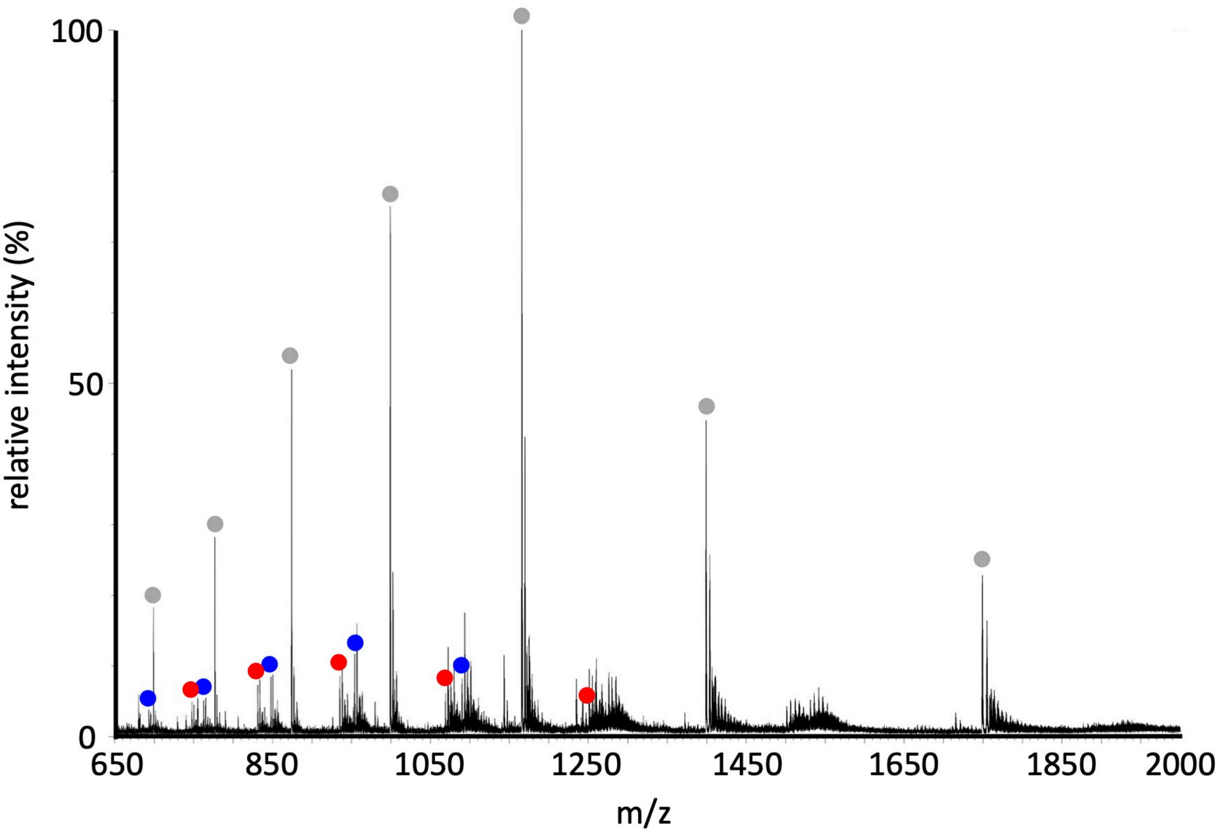

Figure S17. The ESI-mass spectrum of the enzymatic reaction products of hOGG1(K249H) at 15 °C. Blue circle (●): Uncleaved substrate DNA (F-oG23). Red circle (●): The enzymatically oxoG-base-depleted product with the AP-site (F-AP23). Grey circle (●): Complementary strand to F-oG23 (C23). They were observed as multivalent ions with different z (electronic valence).

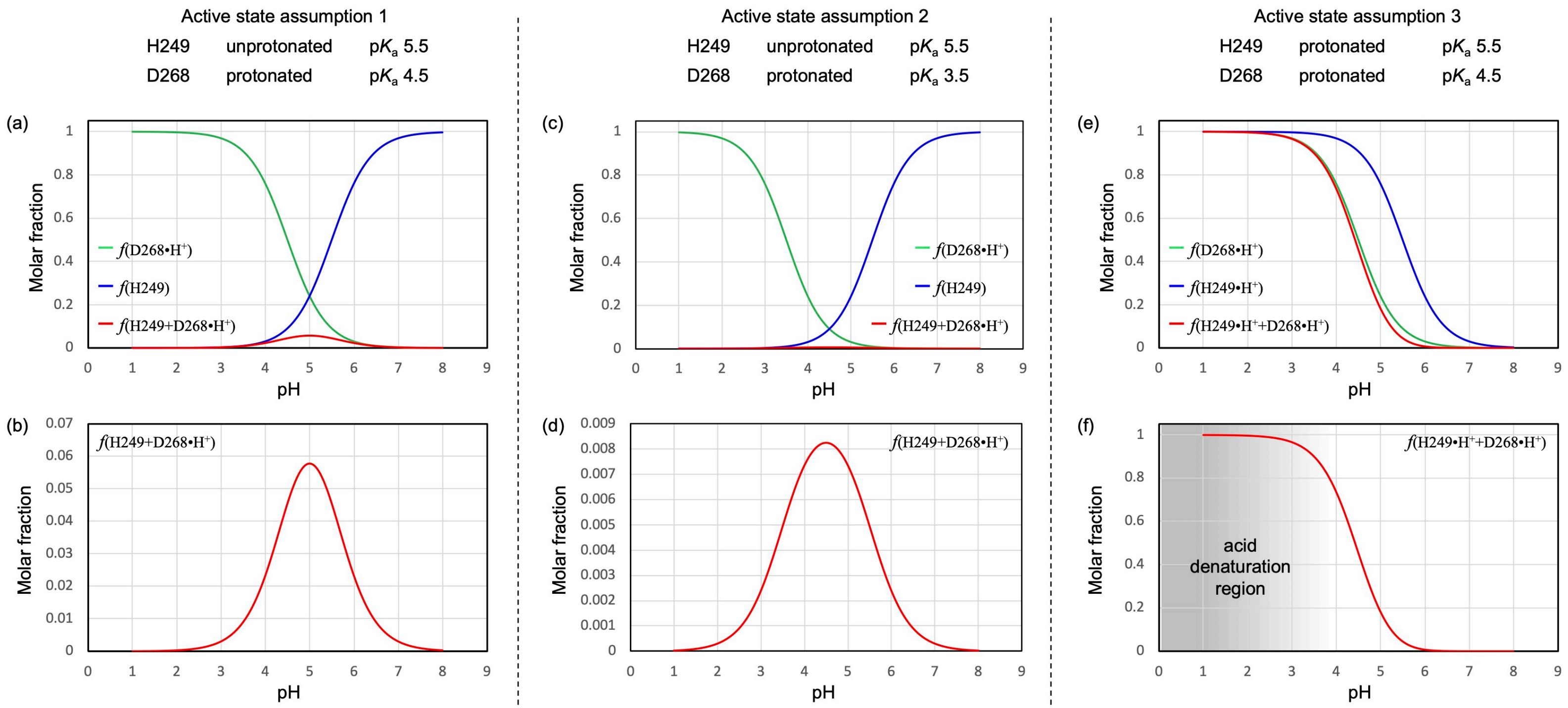

Figure S18. Molar fractions of the protonation states of H249, D268, and the resulting active species. To consider whether H249 is a base (unprotonated form) or not (protonated form), the molar fraction of the active species, f_{active}, was calculated with the equation: $f_{active} = f_{H249} \bullet f_{D268}$ where f_{H249} and f_{D268} are molar fractions of the assumed protonated states of H249 and D268 residues, respectively. The assumed pK_a values and the active forms of the H249 and D268 residues are shown at the top of the panels. Color codes are indicated in the respective panels. Under the conditions in (a) and (b) where unprotonated H249(pK_a 5.5) and protonated D268(pK_a 4.5) is assumed as an active form, the calculated pH of the activity maximum was inconsistent with the experimental one (calculated: pH 5.0, experimental: 4.5). Under the conditions in (c) and (d) where unprotonated H249(pK_a 5.5) and protonated D268(pK_a 3.5) is assumed as an active form, the calculated pH of the activity maximum was consistent with the experimental one (calculated: pH 4.5, experimental: 4.5). However, the molar fraction of the active species was too low (less than 1%). If this is the case for the hOGG1(K249H) mechanism, it will be needed to explain why so a minor state is selected as an active state. On the other hand, under the conditions in (e) and (f) where protonated H249(pK_a 5.5) and protonated D268(pK_a 3.5) is assumed as an active form, the calculated pH-activity curve becomes sigmoidal and can be coherently explained with the equilibrium system.

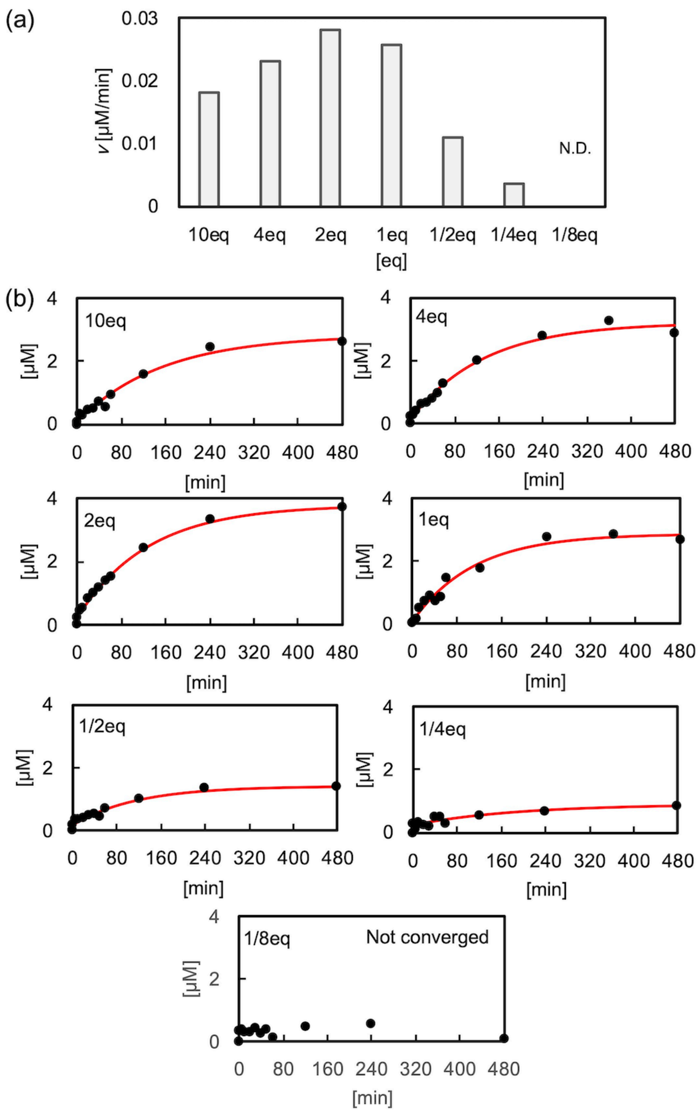

Figure S19. Plot of the reaction rate (v) against the molar equivalency of [enzyme]/[substrate] (a) and the time courses of the enzymatic reactions for the respective molar equivalencies (b). (a) N.D.: not determined. The reaction rate (v) leveled off at almost one equivalent and exhibited the maximum activity at two equivalents. Therefore, two molar equivalents of enzyme/substrate are enough to achieve a single turnover condition where approximately 100% substrate is bound to the enzyme. (b) Time courses of enzymatic reactions for the respective molar equivalencies. The molar equivalencies are indicated in the respective plot areas. The fittings were performed, following the active site titration analysis (12). The fitting was not reliably converged for the condition at 1/8 molar equivalents because of the low reaction rate, and its reaction rate could not be determined.

SUPPLEMENTARY TABLE

Table S1. Experimentally detected and calculated m/z mono-isotopic peaks in the ESI mass spectrum of the enzymatic products in solution

F-AP23 ($C_{248}H_{306}N_{79}O_{149}P_{23}$)

	charge	m/z (obs)	m/z (calc)	err (ppm)
$C_{248}H_{302}N_{79}O_{149}P_{23}$	4	1870.5455	1870.5618	8.7
$C_{248}H_{301}N_{79}O_{149}P_{23}$	5	1496.2441	1496.2479	2.6
$C_{248}H_{300}N_{79}O_{149}P_{23}$	6	1246.6937	1246.7054	9.4
$C_{248}H_{299}N_{79}O_{149}P_{23}$	7	1068.4546	1068.4607	5.7
$C_{248}H_{298}N_{79}O_{149}P_{23}$	8	934.7702	934.7772	7.5
$C_{248}H_{297}N_{79}O_{149}P_{23}$	9	830.7990	830.8012	2.6
$C_{248}H_{296}N_{79}O_{149}P_{23}$	10	747.6137	747.6203	8.9
$C_{248}H_{295}N_{79}O_{149}P_{23}$	11	679.5519	679.5633	16.7

F-oG23 ($C_{253}H_{309}N_{84}O_{150}P_{23}$)

	charge	m/z (obs)	m/z (calc)	err (ppm)
$C_{253}H_{305}N_{84}O_{150}P_{23}$	4	1907.8082	1907.8202	6.3
$C_{253}H_{304}N_{84}O_{150}P_{23}$	5	N.D.	1526.0547	−
$C_{253}H_{303}N_{84}O_{150}P_{23}$	6	N.D.	1271.5444	−
$C_{253}H_{302}N_{84}O_{150}P_{23}$	7	1089.7384	1089.7513	11.8
$C_{253}H_{301}N_{84}O_{150}P_{23}$	8	953.3950	953.4065	12.0
$C_{253}H_{300}N_{84}O_{150}P_{23}$	9	847.3540	847.3605	7.7
$C_{253}H_{299}N_{84}O_{150}P_{23}$	10	762.5165	762.5237	9.5
$C_{253}H_{298}N_{84}O_{150}P_{23}$	11	693.1022	693.1118	13.9

C23 ($C_{223}H_{281}N_{89}O_{132}P_{22}$)

	charge	m/z (obs)	m/z (calc)	err (ppm)
$C_{223}H_{277}N_{89}O_{132}P_{22}$	4	1748.5436	1748.5487	2.9
$C_{223}H_{276}N_{89}O_{132}P_{22}$	5	1398.6279	1398.6375	6.9
$C_{223}H_{275}N_{89}O_{132}P_{22}$	6	1165.3535	1165.3634	8.5
$C_{223}H_{274}N_{89}O_{132}P_{22}$	7	998.7279	998.7390	11.1
$C_{223}H_{273}N_{89}O_{132}P_{22}$	8	873.7610	873.7707	11.1
$C_{223}H_{272}N_{89}O_{132}P_{22}$	9	776.5695	776.5732	4.7
$C_{223}H_{271}N_{89}O_{132}P_{22}$	10	698.8137	698.8151	2.0

Table S2. Soaking conditions for the crystals.

	Crystal 1	Crystal 2	Crystal 3	Crystal 4	Crystal 5	Crystal 6
Time	0 min	30 min	60 min	150 min	24 hours	3 weeks
pH	8.0	4.0	4.0	4.0	4.0	4.0
Temperature (°C)	4.0	4.0	4.0	4.0	4.0	25.0

Table S3. Conditions for the X-ray diffraction data collections

	Crystal 1	Crystal 2	Crystal 3	Crystal 4	Crystal 5	Crystal 6
Beamline	PF BL-5A	PF BL-5A	PF BL-5A	SLS X06SA	PF BL-1A	SLS X06SA
Detector	Pilatus3 S 6M	Pilatus3 S 6M	Pilatus3 S 6M	Pilatus2 MF	Eiger X16M	Eiger X16M
Wavelength (Å)	1.00	1.00	1.00	1.00	1.04	1.00
Oscillation angle (°)	0.25	0.25	0.25	0.2	0.25	0.2
Exposure time (sec)	0.5	0.5	0.5	0.01	0.1	0.01
Collected image (°)	180	180	180	360	180	360
Camera distance (mm)	335.6	335.6	335.6	258.0	116.9	221.0

Table S4. Statistics of the crystallographic data, and structure refinements

	Crystal 1	Crystal 2	Crystal 3	Crystal 4	Crystal 5	Crystal 6
Crystallographic data						
Resolution (Å)	46.20-1.45 (1.47-1.45)	46.12-1.53 (1.56-1.53)	46.18-1.54 (1.56-1.54)	46.26-1.82 (1.86-1.82)	46.18-1.68 (1.71-1.68)	46.19-1.70 (1.73-1.70)
Space group	$P2_12_12_1$	$P2_12_12_1$	$P2_12_12_1$	$P2_12_12_1$	$P2_12_12_1$	$P2_12_12_1$
Unit cell *a, b, c* (Å)	*a* = 61.81, *b* = 69.54, *c* = 87.96	*a* = 61.83, *b* = 69.25, *c* = 88.15	*a* = 61.94, *b* = 69.28, *c* = 88.57	*a* = 62.03, *b* = 69.42, *c* = 88.52	*a* = 61.87, *b* = 69.40, *c* = 88.67	*a* = 61.85, *b* = 69.45, *c* = 88.88
Completeness (%)	99.2 (91.3)	91.5 (46.3)	94.7 (52.5)	98.4 (98.1)	99.9 (98.3)	99.4 (99.5)
I/σ(I)	35.6 (6.6)	22.7 (1.9)	31.4 (2.7)	33.2 (14.1)	8.3 (2.1)	14.2 (5.9)
R_{merge}	0.029 (0.165)	0.052 (0.388)	0.031 (0.150)	0.052 (0.170)	0.143 (1.222)	0.077 (0.617)
$CC_{(1/2)}$	1.000 (0.967)	0.999 (0.815)	1.000 (0.952)	0.999 (0.992)	0.997 (0.514)	0.998 (0.870)
Multiplicity	6.0 (3.3)	5.8 (1.6)	5.6 (1.4)	13.5 (13.9)	6.6 (4.5)	6.6 (6.7)
Structure refinements						
PDB ID	8XWC			8XXG	8XWU	8XXK
R_{work}	0.1546			0.1544	0.1689	0.1630
R_{free}	0.1791			0.1939	0.2135	0.2128
R. M. S.* bond lengths	0.015			0.007	0.015	0.016
R. M. S.* bond angles	1.491			1.050	1.656	1.447

* Root mean square

Table S5. Pseudo-first-order rate constants (k_{obs}) versus pH with their standard deviations (SDs)

pH	k_{obs} / min^{-1}	SD / min^{-1}	C / μM
3.0	2.80 $\times 10^{-5}$	4.06 $\times 10^{-5}$	1.58 $\times 10^{-1}$
3.5	2.24 $\times 10^{-3}$	4.78 $\times 10^{-4}$	2.97 $\times 10^{-1}$
4.0	5.35 $\times 10^{-3}$	1.28 $\times 10^{-3}$	3.27 $\times 10^{-1}$
4.5	7.31 $\times 10^{-3}$	2.44 $\times 10^{-3}$	3.93 $\times 10^{-1}$
5.0	4.70 $\times 10^{-3}$	6.52 $\times 10^{-4}$	3.45 $\times 10^{-1}$
5.5	1.36 $\times 10^{-3}$	6.62 $\times 10^{-4}$	1.96 $\times 10^{-1}$
6.0	4.37 $\times 10^{-4}$	3.49 $\times 10^{-4}$	2.35 $\times 10^{-1}$
6.5	6.83 $\times 10^{-5}$	1.16 $\times 10^{-4}$	1.97 $\times 10^{-1}$
7.0	1.28 $\times 10^{-4}$	1.38 $\times 10^{-4}$	1.22 $\times 10^{-1}$
8.0	1.96 $\times 10^{-4}$	2.55 $\times 10^{-4}$	1.31 $\times 10^{-1}$

SD and C denote standard deviation and the offset value "C" in Eq. S3 (equation *(1)*) due to the non-enzymatic degradation, respectively. Respective k_{obs} values were averaged over four independent experiments under the corresponding pH, and the SD values for the respective pH were calculated. In the pH-k_{obs} plot (Figure 6a), the rate constants (k_{obs}) below pH 4.0 entered a decreasing phase which indicated that another equilibrium system or phenomenon occurred, most probably due to the increasing denaturation of the enzyme under acidic conditions. Therefore, the data at pH 3.0 - 4.0 were not used for the curve fitting (Figure 6a).

Table S6. Pseudo-first-order rate constants (k_{obs}) of hOGG1(K249Q) and hOGG1(K249H) at pH 5.0

	k_{obs} / min^{-1}
hOGG1(K249H)	4.70×10^{-3}
hOGG1(K249Q)	2.64×10^{-5}

SUPPLEMENTARY REFERENCES

1. Nash, H.M., Lu, R., Lane, W.S. and Verdinel, G.L. (1997) The critical active-site amine of the human 8-oxoguanine DNA glycosylase, hOgg1: direct identification, ablation and chemical reconstitution. *Chemistry & Biology*, **4**, 693-702.
2. Fromme, J.C., Bruner, S.D., Yang, W., Karplus, M. and Verdine, G.L. (2003) Product-assisted catalysis in base-excision DNA repair. *Nat Struct Biol*, **10**, 204-211.
3. Nash, H.M., Bruner, S.D., Scharer, O.D., Kawate, T., Addona, T.A., Spooner, E., Lane, W.S. and Verdine, G.L. (1996) Cloning of a yeast 8-oxoguanine DNA glycosylase reveals the existence of a base-excision DNA-repair protein superfamily. *Curr Biol*, **6**, 968-980.
4. Bochevarov, A.D., Harder, E., Hughes, T.F., Greenwood, J.R., Braden, D.A., Philipp, D.M., Rinaldo, D., Halls, M.D., Zhang, J. and Friesner, R.A. (2013) Jaguar: A high - performance quantum chemistry software program with strengths in life and materials sciences. *International Journal of Quantum Chemistry*, **113**, 2110-2142.
5. Jaguar, v. 11.9, Schrödinger. LLC, NY.
6. Impact, v. 9.8, Schrödinger. LLC, NY.
7. QSite, v. 9.8, Schrödinger. LLC, NY.
8. Zhao, Y. and Truhlar, D.G. (2007) The M06 suite of density functionals for main group thermochemistry, thermochemical kinetics, noncovalent interactions, excited states, and transition elements: two new functionals and systematic testing of four M06-class functionals and 12 other functionals. *Theoretical Chemistry Accounts*, **120**, 215-241.
9. Hariharan, P.C. and Pople, J.A. (1973) The influence of polarization functions on molecular orbital hydrogenation energies. *Theoretica Chimica Acta*, **28**, 213-222.
10. Banks, J.L., Beard, H.S., Cao, Y., Cho, A.E., Damm, W., Farid, R., Felts, A.K., Halgren, T.A., Mainz, D.T., Maple, J.R. *et al.* (2005) Integrated Modeling Program, Applied Chemical Theory (IMPACT). *J Comput Chem*, **26**, 1752-1780.
11. Maestro, v. 13.5, Schrödinger, LLC, New York.
12. Porello, S.L., Leyes, A.E. and David, S.S. (1998) Single-turnover and pre-steady-state kinetics of the reaction of the adenine glycosylase MutY with mismatch-containing DNA substrates. *Biochemistry*, **37**, 14756-14764.
